# Intercomparison case study of data-driven reconstructions of a cloud-obscured Saharan dust plume in Europe

**DOI:** 10.1038/s41598-026-55422-y

**Published:** 2026-06-03

**Authors:** Franz Kanngießer, Stephanie Fiedler

**Affiliations:** 1 Institute of Geophysics and Meteorology, Universitsy of Cologne, Pohligstr. 3, 50969 Cologne, Germany; 2 GEOMAR Helmholtz Center for Ocean Research Kiel, Wischofstr. 1-3, 24148 Kiel, Germany; 3 Faculty of Mathematics and Natural Sciences, Kiel University, Christian-Albrechts-Platz 4, 24118 Kiel, Germany; 4 Present Address: Institute of Environmental Physics, Heidelberg University, Im Neuenheimer Feld 229, 69120 Heidelberg, Germany

**Keywords:** Mineral dust aerosol, Observational data, Machine learning, Reconstructing information, Climate sciences, Environmental sciences, Planetary science

## Abstract

On 15 March 2022 an intense, but partially cloud-obscured, Saharan dust plume was transported towards Europe by an atmospheric river. Ten-year statistics of dust plumes co-occurring with cloud cover over Europe highlights that regionally up to 100% of dust plumes are obscured by clouds, which poses challenges for reconstructing dust plumes from satellite images. The European dust case on 15 March 2022 is used to investigate whether data-driven machine-learning techniques for restoring the spatial extent of dust plumes in SEVIRI satellite images can be alleviated by exploiting the rich ground-based data sets in Europe. Satellite images were paired with different combinations of ground-based observation data, stemming from ground-based remote sensing, weather reports and measurements of particulate matter, using a k-nearest neighbours approach. Combining ceilometer and photometer data with satellite images added the most value for restoring the dust plume extent and are recommended for future reconstructions of cloud-obscured dust plumes.

**Supplementary Information:**

The online version contains supplementary material available at 10.1038/s41598-026-55422-y.

## Introduction

Dust transport from North Africa towards Europe occurs during favourable weather (e.g.,^1–5^), e.g., during the occurrence of extra-tropical cyclones over North Africa (e.g.,^6–10^). While African dust plumes typically reach Southern Europe, dust plumes occasionally reach even northern parts, e.g., the British Isles and Scandinavia^1^. Mineral dust transported to Europe has been linked to enhanced melting of Alpine glaciers due to dust deposition^11^, adverse impacts on human health^12^, the disruption of transport and public services^13^, and reduced photovoltaic electricity generation^14^. While intrusions of Saharan dust in Europe have adverse impacts, gauging the full spatial extent of these dust plumes remains challenging, as satellite observations are partially impaired by clouds and numerical forecasts are subject to uncertainty (cf.,^15,16^).

The present study assesses the utility of two different data-driven methods, relying entirely on observations during their application to gain information of the spatio-temporal characteristics of dust plumes over Europe. The purpose of using two methods is the following. First, we illustrate that the reconstruction of intrusions of dust plumes into Europe with the existing machine-learning approach from^17^ is challenging in the case of rare events paired with extensive cloud cover. Motivated by this challenge, we developed a new dust plume reconstruction method taking advantage of multiple ground-based observational datasets in Europe that do not exist with a comparable coverage in North Africa. For building a simple and computationally fast method, we test different data combinations to reconstruct the strongly cloud-obscured dust plume on 15 March 2022 in satellite images to address the question whether adding more and diverse, but partly ambiguous observational datasets is beneficial for the data-driven reconstruction method.

The dust plume on 15 March 2022 was chosen due to its intensity and air-quality impacts in Europe. This plume was associated with the most intense dust event ever recorded in mainland Spain and Portugal^18^ and marked the beginning of several Saharan dust intrusions in Europe (cf.,^19^). On 15 March 2022, hourly values of particulate matter with a maximum diameter of $10\,\mu$m (PM10) exceeded $700\,\mu$g m$\vphantom{0}^{-3}$ in Malága^20^ and 24h averages of PM10 throughout the Iberian Peninsula exceeded $70\,\mu$g m$\vphantom{0}^{-3}$, with a record of $3069\,\mu$g m$\vphantom{0}^{-3}$ in mainland Spain^18^. For comparison, European air quality legislation defines the limit of daily mean PM10 at $50\,\mu$g m$\vphantom{0}^{-3}$^21,22^. On 2022-03-17, the dust plume reached Hungary^23^, and the day after it reached Finland^24^. Furthermore, the 2022-03-15 dust event is linked with the transport of radionuclides to Western Europe, which did not pose a public health risk^20,25^. According to an analysis of Saharan dust events in Eastern Spain between 1998 and 2002, March and October display a high frequency of dust days, second only to the summer^26^. In recent years (2020–2022) Saharan dust intrusions over the western Euro-Mediterranean increased sharply during February and March, compared to the earlier years of 2003–2019^5^, making the March event additionally interesting for our study.

The case of 15 March 2022 was also interesting from the perspective of the meteorological processes at play involving an atmospheric river. Atmospheric rivers are elongated bands of high water vapour content (e.g,^27^), commonly associated with clouds and precipitation (e.g.,^27–29^). Although 78% of atmospheric river events over northwest Africa are associated with dust transport towards Europe, only 18% were strong or extreme dust plumes between 1980 and 2020^4^. A considerable amount of atmospheric river landfalls occur in northwestern Africa^27,29^, advecting warm, moist air from the Atlantic Ocean^4,29^. These events cause dust emission^29^ due to their strong near-surface winds^28^.

Observations of dust plumes with a high temporal resolution, which simultaneously yield large spatial coverage, as provided by geostationary satellites, poses challenges. In case of dust transported to Europe, the Spinning Enhanced Visible and Infrared Imager (SEVIRI) onboard the Meteosat Second Generation (MSG) satellites provides observations. SEVIRI passively observes the radiance in 12 different spectral channels and a broadband channel with a temporal resolution of 15 minutes^30^. However, transported dust is frequently co-occuring with clouds^4^, especially expected during atmospheric rivers as described above. It implies, clouds partially or fully obscure the dust plume in satellite observations.

Cloud-induced or other gaps, are common in such datasets and techniques for reconstructing the variables of interest exist. Earlier studies on gap filling frequently focus on data, which is independent of atmospheric motion including cloud patterns, such as land cover^31–33^, phytoplankton^34,35^, sea surface temperature^36,37^, land surface temperature^38,39^ and evapotranspiration^40^. Other gap filling studies considered gaps in stream-function observations^41^, temperature anomalies^42^ and extremes^43^, and soil moisture^44^. Furthermore, gaps in aerosol information, which is subject to atmospheric circulation, have been removed^17,45^.

Several geophysical reconstruction methods exist that leverage trends, established associations between different variables and/or spatio-temporal patterns (e.g.,^17,34,40,44,45^). A classical geostatistical gap restoration approach is kriging^46,47^, which uses linear interpolation/extrapolation and employs radial covariances from available observations^48^. Kriging has demonstrated good performance in restoring missing values close to known values, but resulting in over-smooth and even unsuitable values^34,42,45,48^. More refined kriging approaches like spatio-temporal kriging require careful adaptation and testing for each region of interest^34^. Kriging-based approaches work best for patterns characterised by weak gradients and without categorial patterns^34^. Another well-established geostatistical approach are data interpolating empirical orthogonal functions (DINEOFs). DINEOF is an iterative approach using spatial and temporal patterns of variability encoded in empirical orthogonal functions (EOFs) and can be considered a well-performing general purpose method^34,35^. It is especially suited for large areas characterised by slow rates of change and smooth gradients^35^. DINEOF requires a time series long enough to capture the underlying variability, but too long time series may suppress the transient variability and only reproduce large-scale patterns, but fail to reproduce patterns at smaller scales^35^.

There are different machine learning techniques used for gap filling, that effectively perform regression tasks. One subset of these techniques, Random Forests and gradient boosting, are based on binary decision trees^38,39,44,45^. These machine-learning-based regression techniques further include support vector machines (SVMs), which are based on optimisation theory. SVMs are commonly used to perform reconstructions using linear regressions, but similarly to decision-tree-based regressions they can be used for non-linear regressions^39,44^. These regression approaches infer relationships between the target variable and multiple other variables. By extension, these approaches typically require gap-free associated datasets to reconstruct gaps in the target variable.

There are also reconstruction approaches based on deep learning methods. In particular, this includes methods based on generative adversarial networks (GANs) (e.g.,^32,33,36,37^) and convolutional neural networks (CNNs) and architectures derived from CNNs (e.g.,^17,31,35,41–43^). GANs have been successfully employed in geophysical gap-filling studies for reconstructing large-scale features. They typically require smaller training datasets than CNNs or CNN-derived architectures (e.g.,^17,31,35–37,42^). Training and/or validating both GAN- and CNN-based networks for reconstruction purposes often build upon pairs of gap-free data and gap-containing data. These pairs are created by masking gap-free target variables in another, originally gap-free area (e.g.,^31,35,36^) or masking related variables such as reanalysis or model output variables (e.g.,^17,41–43^). Applying masks from one area to data from another area requires the data and the gaps to be independent, which is not the case for cloud-induced gaps in atmospheric variables. GAN-based reconstructions appear realistic, but do not necessarily match a ground truth^36,42,49^. This may be disadvantageous for classification tasks, to which reconstructing the spatial patterns of dust plumes ultimately amount to.

In this study, we apply an in-painting method, which we successfully employed for dust plume reconstructions in North Africa^17^. This method, which is based on partial convolutional neural networks (PCNNs) relies on common patterns of dust plumes and the associated cloud masks for reconstruction. The in-painting method’s success is unclear in the case of rare and strongly cloud contaminated cases like expected for cases of dust outbreaks towards Europe. We investigate the prevalence of dust plumes transported to Europe and their co-occurrence with clouds, using reanalysis data. Europe’s rich ground based observation network is here an ideal opportunity to evaluate this method under rare cases and additionally compare the quality against a newly presented method. This new method combines satellite with ground observations and is computationally efficient. Unlike the common reconstruction strategies introduced above, the newly presented method does not require long-term information, instead the reconstruction is solely based on the available information at any given time of interest.

The new method combines different ground-based datasets, specifically observations of the attenuated backscatter from automated lidars and ceilometers (ALC) provided by the European Meteorological Network (EUMETNET) (cf.^50^), aerosol optical depth and Ångström exponent from photometer observations provided within the framework of the Aerosol Robotic Network (AERONET) (cf.^51^), reports of significant weather as provided by regular routine observations from automated and manned stations provided via the Integrated Surface Database (ISD) (cf.^52^), and in-situ measurements of particulate matter (PM) provided by the European Environment Agency (EEA) (cf.^22^). Specifically, we inferred the presence and if possible absence of dust at the respective observational stations or in case of satellite images, in the respective pixel, and performed a computationally inexpensive classification based on a k nearest neighbours (kNN) approach^53,54^, which does neither require long-term time series of variables nor spatial patterns of single or several variables. Each of these observational datasets comes with specific limitations regarding the interpretability of the available information, as the data are partly ambiguous. We therefore test different combinations of data sets for reconstructing the dust plume extent during rare events with large-scale cloud cover.

This manuscript is organized as follows. We first provide an analysis of co-occurrences of dust plumes with clouds in Europe in “Climatology of dust plumes in Europe” section to provide a climatology context. “Machine-learning-based in-painting” section presents results for the PCNN-based reconstruction on 2022-03-15 followed by the kNN-based method in “Data-driven kNN reconstructions” section, and additional dust plume cases in “Further test cases” section. A discussion and conclusion are presented in “Discussion” and “Conclusions” sections, respectively. Details for data and methods are included in “Methods” section. Finally, “Synoptic situation during 2022-03-15” section provides brief synoptical context for the dust plume on 2022-03-15.

## Results

### Climatology of dust plumes in Europe

**Fig. 1:**
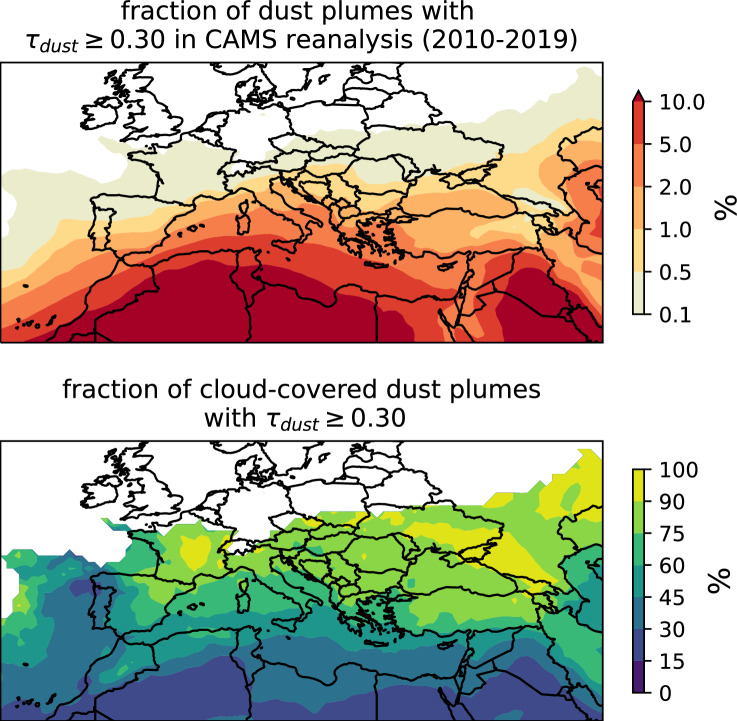
The top panel shows the spatial distribution of the dust plumes in percent of time during ten years of CAMS reanalysis (2010-2019), during which a dust aerosol optical depth of at least $\tau _\textrm{dust}=0.30$ was reached. The lower panel shows the fraction of dust plumes from the corresponding upper panel covered by clouds. Maps were created using the Python package Cartopy (version 0.24.1, https://scitools.org.uk/cartopy/docs/v0.24/index.html)^57^.

Within Europe, ground-based observations are ubiquitous, however, the occurrence of dust plumes decreases with increasing distance from the Mediterranean (cf.^2,4^). To quantitatively estimate the number of dust plumes over Europe, we used the CAMS reanalysis dataset. This dataset is produced by the Copernicus Atmospheric Monitoring Service and provides a global reanalysis of the atmospheric composition^55,56^. From CAMS reanalysis, we could infer a decreasing number of dust plumes with increasing distance to the Mediterranean, as illustrated in Fig. 1. This figure indicates how frequent dust plumes occur and how often they co-occur with clouds between 2010 and 2019. The upper panel shows the fraction of time steps within this ten-year period, during which in an individual grid box a dust aerosol optical depth of at least $\tau _\textrm{dust}=0.30$ was reached. The aerosol optical depth quantifies spectral extinction due to aerosols according to the Beer-Lambert-Bouguer law (cf.,^51^). Dust aerosol optical depth then quantifies the extinction due to mineral dust aerosol specifically. In entirely dust-free conditions, $\tau _\textrm{dust}$ would take values of 0. Strictly speaking, there is no upper limit, but the largest value in the analysed ten-year period north of 36$\vphantom{0}^{\circ }$N (i.e., the Strait of Gibraltar) was $\tau _\textrm{dust}=2.06$. For example, over the Iberian Peninsula $\tau _\textrm{dust}$ reaches values of at least 0.3 only in 0.5–2% of time steps, but only for between 0.1–0.5% of time steps in most of France. The lower panel of Fig. 1 shows the fraction of dust plumes, which are covered by clouds. To estimate the fraction of dust plumes obscured by clouds, we employ the total cloud cover, as reported within the CAMS reanalysis, which takes values between 0 and 1, which corresponds to entirely cloud-free conditions and a completely overcast sky, respectively. If the total cloud cover within a grid box reaches a value of 0.5 or more, we consider the grid box to be cloud-covered. Not only occurred most dust plumes in North Africa, but the fraction of cloud-obscured dust plumes there was also the lowest. Dust plumes occurring on the northern Iberian Peninsula, France, and northern Italy are to, at least, 75% obscured by clouds and dust plumes with $\tau _\textrm{dust}\ge 0.30$ in central France were even to 90 - 100% covered by clouds. Fig. S1 shows the values for optically thinner dust plumes, which are more frequent and extend farther north. Specifically, on the southern and central Iberian Peninsula dust plumes with $\tau _\textrm{dust}\ge 0.15$ occur between 2.0–10.0% of the time and to at least 0.5% of the time in France with up to 2.0–5.0% along the France’s Mediterranean coast.

The annual variability of the zonal mean dust occurrence and the associated cloud co-occurrence can be inferred from Fig. S2. The area for which the zonal mean was calculated, was bound by $20^{\circ }$W and $52^{\circ }$E for the CAMS reanalysis grid boxes centred between $36.75^{\circ }$N and $45.0^{\circ }$N. Fig. S2 reveals, that most dust plumes between 2010 and 2019 occurred in April and May, while the least dust plumes occurred during November and December. The fraction of dust plumes co-occurring with clouds increased in general with increasing latitude.

The relative low number of dust plumes (see Fig. 1) transported to Europe may present challenges for training an artificial neural network (ANN) due to the limited size of the training samples. In other words, the number of events, even when considering ten years of reanalysis, could be too low, to successfully reconstruct plumes of dust transported to Europe. To address the challenges in reconstructing dust plumes transported to Europe, we focused on the dust plume of 2022-03-15, which co-occured with clouds.

SEVIRI products include false-colour images. These images are created by assigning observed brightness temperatures and differences of brightness temperature to the red (R), green (G), and blue (B) channels, into which each colour in RGB colour space can be decomposed into. With the help of these false-colour images different atmospheric features, such as cloud types, air masses, trace gases, volcanic ash, and mineral dust can be identified^58^. For the identification of mineral dust, EUMETSAT provides the Dust RGB, in which dust plumes are shown in magenta. In the morning hours of 14 March 2022, as full resolution Dust RGB images by EUMETSAT indicate, dust was lifted in the south of Algeria and at the Atlas Mountains along the Moroccan-Algerian border (not shown). The dust was subsequently transported towards Europe by an atmospheric river and reached the Iberian Peninsula around 14:15-14:45 UTC. In the following hours, dust was transported further north and east, across the Bay of Biscay and over France.

**Fig. 2:**
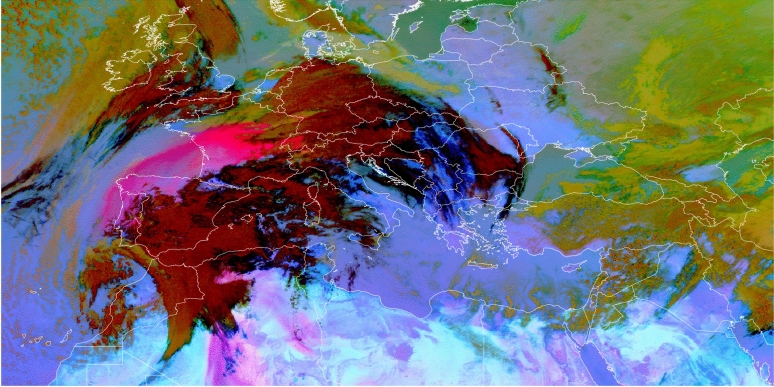
Full resolution Dust RGB image during 2022-03-15, 12:00 UTC for the region of interest. For better guidance, coastlines and borders were overlayed. Image provided by EUMETSAT^59^.

The dust plume observed on 2022-03-15 (see Fig. 2) was unusual regarding its spatial extent, especially its reach north, and representative of the climatological mean for the co-occurring cloud cover. On 2022-03-15, 12 UTC and in the region bound by the latitudes of 36$\vphantom{0}^\circ$N and 60$\vphantom{0}^\circ$N, and the longitudes of 20$\vphantom{0}^\circ$W and 52$\vphantom{0}^\circ$E, CAMS reanalysis shows that 73% of native resolution grid boxes with $\tau _\textrm{dust}\ge 0.15$ are covered by clouds. When considering grid boxes with $\tau _\textrm{dust}\ge 0.30$, 75% are covered by clouds. When considering the 10 year average for March based on CAMS reanalysis of 70%, and 73% respectively, these conditions represent a typical situation (see Fig. S2). As in “Climatology of dust plumes in Europe” section, a grid box is considered to be covered by clouds, when the total cloud cover within in the CAMS reanalysis was at least 0.5.

### Machine-learning-based in-painting

One reconstruction method relied on machine-learning-based in-painting, specifically the climatereconstructionAI algorithm (CRAI)^42,60^, which was successfully used to reconstruction cloud-obscured dust plumes across North Africa^17^. As the CRAI algorithm is based on a neural net with partial convolutions, the resulting reconstructions are referred to as PCNN-based. The CRAI algorithm was trained on three different datasets:

- CAMS dust aerosol optical depth (AOD) reanalysis with a cloud mask obtained from the temporally matched SEVIRI cloud mask product between 2020-09-01 and 2021-12-31, denoted as “CAMS+SEVIRI”,
- CAMS dust AOD with cloud masks obtained from the CAMS total cloud cover variable for all dust plumes north of 36$\vphantom{0}^{\circ }$N with at least ten grid boxes in native CAMS resolution with $\tau _\textrm{dust}>0.3$, denoted as “CAMS clim.”,
- as the training dataset above, but with at least 100 grid boxes in CAMS native resolution with $\tau _\textrm{dust}>0.3$ north of 36$\vphantom{0}^{\circ }$N, denoted as “CAMS clim. (extremes)”.

See “Machine-learning-based image in-painting” for more detailed information on the PCNN-based reconstruction and the training datasets. Fig. 12 provides a brief schematic representation of the reconstruction method, including training approach and input data and compares it to the data-driven method, of which the results are presented in “Data-driven kNN reconstructions” section.

**Fig. 3:**
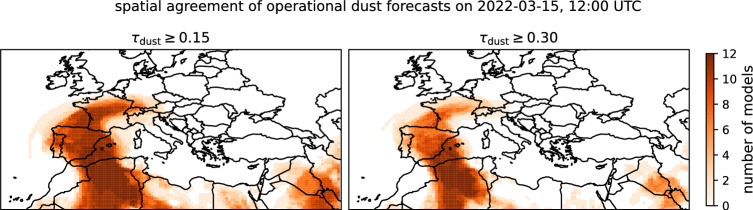
Spatial agreement of individual operational forecast models in the forecast ensemble provided by the WMO Dust Regional Center Barcelona on 2022-03-15 at 12:00 UTC. The respective models and the lead time of the forecast are given in Tab. 1. The shading indicates for how many models a dust aerosol optical depth of $\tau _\textrm{dust}\ge 0.15$ (left) and $\tau _\textrm{dust}\ge 0.30$ (right) was forecasted for each grid box. Maps were created using the Python package Cartopy (version 0.24.1, https://scitools.org.uk/cartopy/docs/v0.24/index.html)^57^.

We evaluated the performance of the reconstruction of the dust plume by calculating the Sørensen-Dice similarity coefficient (*SC*)^61,62^, the Matthews correlation coefficient (*MCC*)^63,64^,and the Hausdorff distance^65,66^ between the reconstructions and the dust predictions from twelve different dust numerical forecasts provided by the WMO Dust Regional Center Barcelona^67^. For an overview over the individual models see Tab. 1. Dust plume spatial patterns were obtained from numerical forecasts by using the dust AOD forecast. If $\tau _\textrm{dust}$ reaches at least 0.15 and 0.30 the gridbox is considered to be dust-containing, otherwise we classify it as dust-free. The models differ in their forecasts of dust plume locations. To fully capture the spatial ensemble spread, as well as the ensemble agreement, Fig. 3 indicates the number of models forecasting a dust plume with $\tau _\textrm{dust}\ge 0.15$ (left) and with $\tau _\textrm{dust}\ge 0.3$ (right). White indicates no model forecasting a dust plume at a given location, while dark orange indicates the full ensemble forecasting a dust plume at a location. For the plume with $\tau _\textrm{dust}\ge 0.15$, the models show generally high agreement, with the exact boundaries being subject to some uncertainty. Conversely, the models do not agree on the magnitude of $\tau _\textrm{dust}$, as only half the ensemble forecasts a dust plume with $\tau _\textrm{dust} \ge 0.3$ over the Iberian Peninsula, the Gulf of Biscay, and France.

The true shape and border of the dust plume may be considered inherently uncertain, as plume information inferred from reanalysis differs between reanalysis datasets^17^. To account for this inherent uncertainty and to increase robustness of the evaluation, the reconstructions are evaluated against the individual forecast of the operational dust forecast ensemble.

**Fig. 4:**
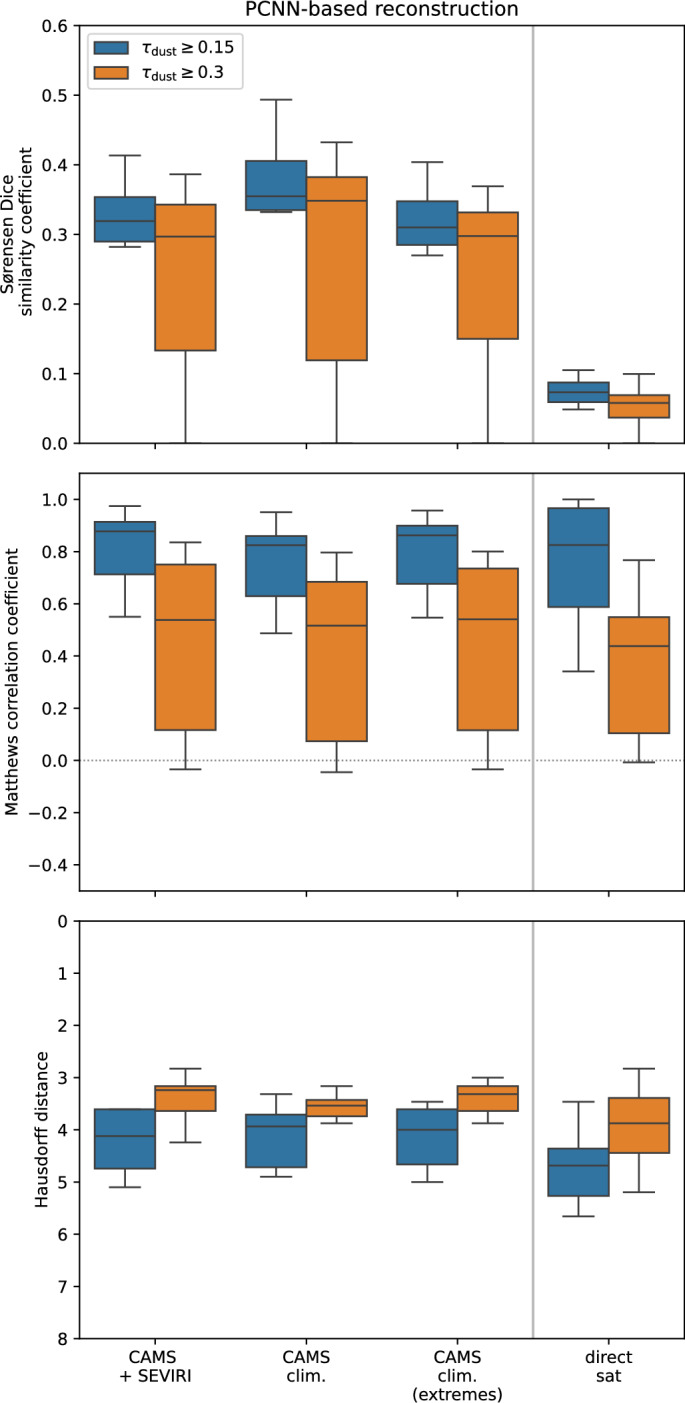
Sørensen-Dice similarity coefficient (SC, top), Matthews correlation coefficient (MCC, centre), and Hausdorff distance (bottom) at 2022-03-15, 12 UTC between the PCNN-based reconstruction compared to spatial patterns of dust plumes obtained from numerical forecasts provided by the WMO Dust Regional Center Barcelona (see Tab. 1 for all models) Depending on the numerical model, the numerical forecast product pertains either to the analysis or the 12h forecast. The categories on the x-axis pertain to the different training strategies of combining cloud patterns obtained from the SEVIRI operational cloud mask with dust information from CAMS reanalysis (CAMS+SEVIRI) and combining both dust plumes and cloud patterns from CAMS reanalysis moderate (CAMS clim.) and large (CAMS clim. (extremes)) dust plumes. For comparison, the direct or non-reconstructed satellite observations are shown (indicated by “direct sat”).

During the evaluation we further restricted the region of interest, which is then bound by the latitudes of 36.09$\vphantom{0}^{\circ }$N and 60$\vphantom{0}^{\circ }$N, and the longitudes of 20$\vphantom{0}^{\circ }$W and 27.25$\vphantom{0}^{\circ }$E. Fig. 4 shows the calculated values of SC, MCC, and Hausdorff distance for the PCNN-based reconstructions for the three different training datasets with respect to the references, i.e. the spatial extent of forecasted dust plumes. For comparison, we also show the SC calculated from the direct, i.e. non-reconstructed, SEVIRI observations. The SC evaluates the overlap between two sets and penalises non-overlap, regardless of the distance. The MCC describes the quality of binary classification tasks. In order to achieve a high MCC ($MCC \rightarrow 1$) the majority of positive and negative cases must be classified correctly^64^. The Hausdorff distance describes the mismatch between two sets or images, with a value of 0 indicating a perfect agreement between the images. It is robust against small deviations, as it considers the distance rather than the overlap^65^ For a more detailed overview over the evaluation metrics, please see “Evaluation metrics” section. Compared to the direct observations, the PCNN-based reconstructions added information, as reflected by the higher values of SC. Both the MCC and the Hausdorff distance display less differences between the direct observations and the three different reconstructions.

**Fig. 5:**
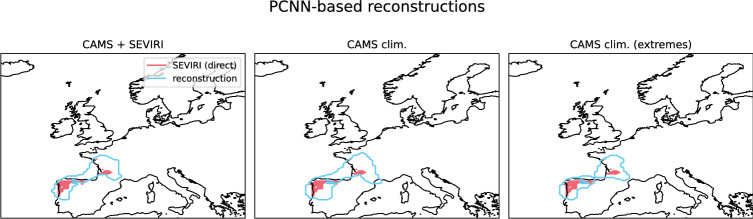
Spatial extent of the dust plume at 2022-03-15, 12 UTC from direct SEVIRI observations and PCNN-based reconstructions. The different panels indicate the different underlying training datasets. Maps were created using the Python package Cartopy (version 0.24.1, https://scitools.org.uk/cartopy/docs/v0.24/index.html)^57^.

To aid the interpretation of these values, Fig. 5 shows the spatial extent of the direct or non-reconstructed SEVIRI observations and from PCNN-based reconstructions. PCNN-based reconstructions succeeded in combining disconnected observed dust plumes into a larger plume (Fig. 5). Still, as already evident from Fig. 4, these PCNN-based reconstructions differed from the numerical dust forecasts. Specifically, the CRAI algorithm failed to reconstruct, cloud obscured dust in the southern part of the Iberian Peninsula, whereas, the entire forecast ensemble agreed on the presence of dust. For the cloud cover as reported by ERA5 reanalysis, see Fig. 14. While the MCC and the Hausdorff distance are generally suitable tools for evaluating classification tasks and image similarity^63,65,66^,they do not successfully quantify the differences between the direct SEVIRI observations and reconstructions with respect to the numerical dust forecasts. The comparison of Figs. 5 and 3 suggests, that the direct observations do not yield false positives when compared to numerical forecasts with $\tau _\textrm{dust}\ge 0.15$ as a reference. The lack of false positives, in this case increases the MCC (see Eq. 3).

The “CAMS+SEVIRI” training dataset, which was constructed following^17^, did not explicitly consider dust plumes transported to Europe and may lack a sufficient number of plumes extending across the Pyrenees. To increase the number of dust plumes transported to Europe in by the training dataset, CAMS-derived cloud masks were combined with the CAMS dust AOD reanalyses. Note, however, that the cloud coverage as inferred from SEVIRI operational cloud mask and the total cloud cover from reanalysis is not necessarily identical (cf.^68^). Using reanalysis to include cases of dust transported to Europe (“CAMS clim.”) did yield small improvements in terms of SC compared to training on the combination of CAMS reanalysis fields with SEVIRI cloud masks (“CAMS+SEVIRI”). Considering only cases of large dust plumes (“CAMS clim. (extremes)”), which are defined as at least 100 grid boxes in CAMS native resolution north of 36$\vphantom{0}^{\circ }$N with $\tau _\textrm{dust}>0.3$, did result in marginally poorer reconstructions. All in all, extending the training dataset to include more cases of north-ward dust transport hardly improves reconstructions. The PCNN-based reconstruction adds value compared non-reconstructed direct SEVIRI observations, but fails to reconstruct dust, especially further south on the Iberian Peninsula.

### Data-driven kNN reconstructions

We developed a new reconstruction method leveraging the dense observational network across Europe to detect dust underneath clouds. Different ground-based observational datasets were combined with dust-plume information extracted from gray-scaled SEVIRI Dust RGB images to restore the spatial extent of the dust plume. To that end, we used a k-nearest neighbours (kNN)^53,54^ classification. The k-nearest neighbour classification assigns a class to each point, based on scores calculated from the classes of the *k* nearest neighbours, where *k* takes a pre-defined integer value^53,54^. The values of these *k* nearest neighbours can be either weighted uniformly or by the inverse distance to the point of interest, i.e. with closer points having a higher weight in the calculation of the scores. The resulting reconstructions are referred to as kNN-based reconstructions. As introduced in “Introduction” section, we incorporated passive sun photometer observations provided by the Aerosol Robotic Network (AERONET)^51,69^, observations from automated lidars and ceilometers (ALC)^50^, weather observations from the Integrated Surface Database (ISD)^52^ provided by NOAA, and in-situ observations of particulate matter collected from air quality stations and provided by the European Environment Agency (EEA)^22^. We tested three different types of weighting the observations used as input for the kNN reconstruction. In case of uniform weighting each observation was assigned the same weight. For distance weighting a higher weight was assigned the closer an observation was to the grid point to be reconstructed. For direction-dependent distance weighting, we introduced an alternative distance metric, which takes the wind direction into account. Wind directions were taken from the SEVIRI atmospheric motion vector (AMV) product^70^. More details on the method are given in “Reconstruction by k-nearest neighbour classification” section. The ground-based observational datasets, their limitations, and thresholds used to infer the presence of dust are presented in “Ground-based observations” section. A brief schematic of the method and a comparison with the PCNN-based method, of which the results were introduced in “Machine-learning-based in-painting” section, is given in form of Fig. 12.

**Fig. 6:**
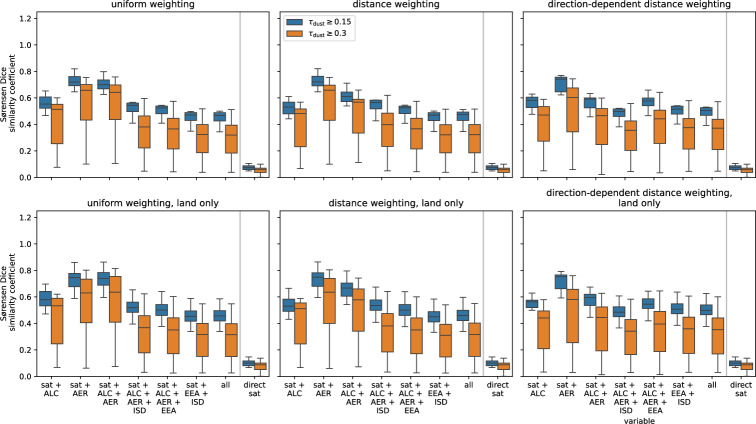
SC of data driven reconstructions with respect to spatial patterns from the numerical forecast ensemble. The different classes represent the different combinations of observational datasets. The left column shows results for applying uniform weighting in the kNN classification. The middle column displays results for kNN classifications, with points weighed according to the Euclidean distance. Results for direction-dependent distance weighting using the semi-latus rectum of an ellipse as a distance metric are shown in the right column. Directions were inferred from the SEVIRI AMV product. The top row, shows the SC for the entire domain. The bottom row considers only grid boxes over land, since all observational datasets were land-based. For comparison, the right-most entry in each panel shows the SC of dust plumes directly extracted from SEVIRI dust RGB images with respect to the numerical forecasts.

Combining satellite observations with ground-based remote sensing observations, specifically AERONET (denoted as AER), and combined AERONET/ALC observations, yielded the highest overlap between reconstructions and numerical forecasts with $SC \sim 0.7-0.8$, as can be seen in Fig. 6. Only incorporating ALC observations as additional data source, resulted in lower values of the SC compared to the other two remote sensing datasets. Adding further data sources, such the ISD weather observations or the EAA in-situ observations of PM10, reduced the SC. It also resulted in an implausibly large spatial extent of the reconstruction, as evident from Fig. S6. However, compared to the direct satellite observations, the SC of all kNN reconstructions was consistently higher. Further, limiting the assessment of the reconstruction to plumes over land surfaces, yielded small improvements ($\Delta SC \ge 0.03$) for reconstructions based on the combination of SEVIRI observations with ALC and AERONET observations. For the remaining combinations of observational data, the changes in SC were smaller, when only considering reconstructions over land surfaces.

Assessing the kNN-based reconstructions with the help of the MCC indicated the best performance when combining the satellite observations with ground-based remote sensing observations (see Fig. S4). As for the SC as evaluation criterion, the MCC for reconstructions was lower, when observations reported by the EEA or within the ISD were incorporated. In general, these reconstructions yield MCCs between -0.2 and 0.2. Values of $MCC \approx 0$ are akin to random guessing^64^. When considering direction-dependent distance weighting, the reconstructions performed worse in terms of MCC, except for satellite observations combined with ground-based remote sensing and EEA observations. Notably, the reconstruction incorporating only AERONET as an additional data source resulted in the highest MCC, but the MCC for direction-dependent distance weighting was still lower than for uniform and distance weighting. Evaluating the reconstructions in terms of Hausdorff distance (Fig. S5), showed the same patterns, as seen by using the MCC.

Each dataset had unique challenges in the interpretation. ALCs can detect lofted aerosol layers, but do not identify the aerosol type^50^. Photometer observations retrieved aerosol optical depth and size distribution information for the atmospheric path between the instrument and the sun and therefore require direct sunlight^51^. Reports of significant weather may report dust, but attribution to the region of origin is challenging. Further, current and past weather is reported according to priority. Weather assigned a higher priority supersedes weather with a lower priority, e.g. precipitation observations have a higher priority than most dust observations. There is also no code for reporting the absence of dust^52^. Measurements of particulate matter do not distinguish between aerosol types, provide only in-situ data, and, thus, no information about the entire column. In other words, if the particulate matter measurements indicate absence of dust at the surface, it does not preclude lofted dust is not occurring^22^.

**Fig. 7:**
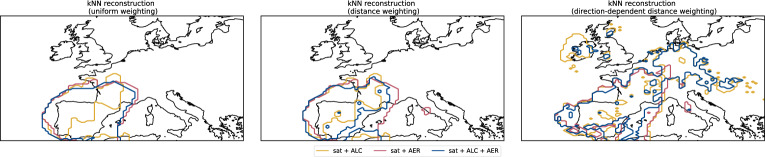
Spatial patterns of dust plumes obtained from kNN reconstructions. The panels indicate different weighting of the data points, i.e. uniform weighting (left), distance weighting (centre), and direction-dependent distance weighting (right). Colours indicate the observational datasets used for the reconstruction. Only results for the three best-performing combinations of observational data sets are shown. The results from the remaining data sets are shown in Fig. S6. Maps were created using the Python package Cartopy (version 0.24.1, https://scitools.org.uk/cartopy/docs/v0.24/index.html)^57^.

Fig. 7 shows the spatial patterns of the best-performing kNN-based reconstructions. For the remaining reconstructions, see Fig. S6. For comparison, the corresponding numerical forecasts of dust plumes are shown in Fig. 3. The spatial patterns indicate, that incorporating ISD and EEA data was, despite measures to reduce ambiguities, prone to introduce false positives. Furthermore, both EEA and ISD data provide no conclusive indications about the absence of dust (or other lofted aerosol plumes). Hence, the reconstructions were insufficiently constrained regarding dust-free situations. Similarly, the lack of ground-based observations over sea surfaces may yield further insufficiently constrained reconstructions. Limiting the reconstructions to plumes above land surfaces only, i.e., regions with ground-based observations, improved the SC values marginally on 2022-03-15 at 12 UTC, as can be seen in Fig. 6. Considering direction-dependent distance weighting, did have diametral effects on the spatial pattern of the reconstructed dust plume, when visually comparing it to the numerical forecasts.

**Fig. 8:**
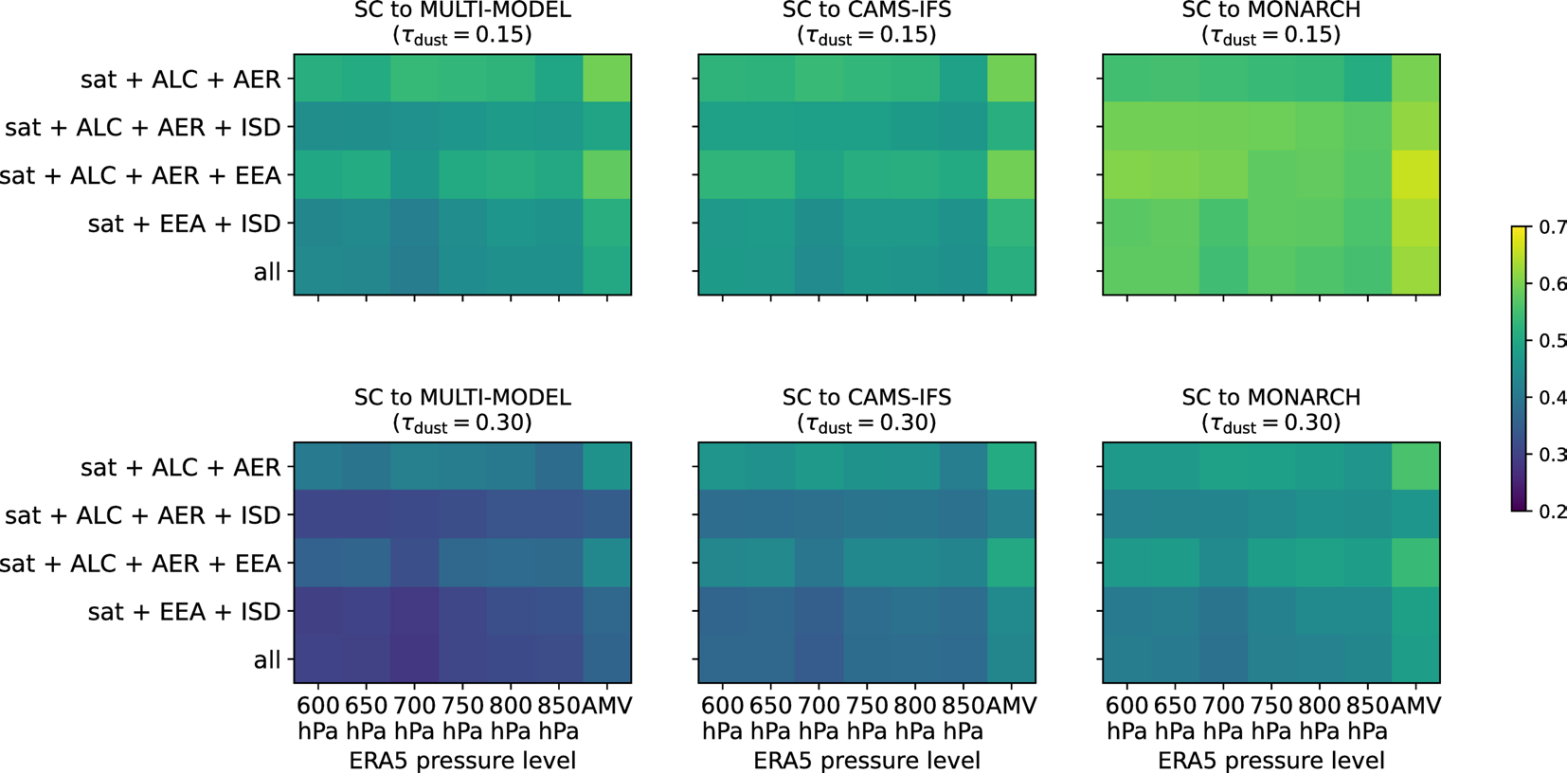
Comparison of SC between direction-dependent kNN reconstructions with respect to the median forecast (MULTI-MODEL, left), CAMS-IFS (centre), and MONARCH (right) with $\tau _\textrm{dust}\ge 0.15$ (top) and $\tau _\textrm{dust}\ge 0.30$. Each panel shows the different combinations of observational data set on the y-axis. The ERA5 pressure level, from which the wind information was taken, are given on the x-axis. Wind-directions taken from the SEVIRI-AMV product are indicated with AMV.

To gauge the effect of the source of directional data, we compared the results obtained when using the SEVIRI AMV product^70^ to results when using wind directions taken from ERA5 reanalysis of the horizontal components of the wind vector at different pressure levels between 600 and 850 hPa (cf., Fig. S3)^71,72^. The SEVIRI AMV product is obtained by tracking clouds and water vapour features (cf.,^73,74^). As before, we assessed the reconstruction by calculating the SC between the reconstructions and numerical dust forecast. The results are presented in Fig. 8. Here, we focused on forecasts from CAMS-IFS^75,76^, MONARCH^77,78^, and the multi-model median forecast over the available forecast ensemble from up to 14 models, referred to as MULTI-MODEL^16,67^. With respect to the three numerical forecast models, CAMS-IFS, MONARCH, and the ensemble median forecast MULTI-MODEL, the SC of direction-dependent distance weighting using directions incorporated from the SEVIRI AMV product was higher than for reconstruction incorporating directions inferred from ERA5 horizontal wind vectors at the different pressure levels. Thus, replacing directional information obtained from the interpolated SEVIRI AMV product with information obtained from ERA5 reanalysis did not improve, but decreased the quality of the directional-dependent distance weighting. As the dust plume was transported by an atmospheric river (see Sec. Synoptic situation during 2022-03-15), water vapour features were expected to indirectly encode relevant dust plume information.

### Further test cases

To further assess the different reconstruction techniques, additional test cases were selected. Potential test cases were identified using English-language news reports of dust plumes across Europe. After checking for the presence of a dust plume by visually inspecting Dust RGB images in native resolution for dust plumes during day-time conditions, test cases were selected, if ground-based observations and operational numerical forecasts from several models are available. Additional reconstructions were performed for the following test cases: 2022-04-23 15:00 UTC, 2024-06-08 09:00 UTC, and 2024-06-19 12:00 UTC. The kNN-based reconstruction combine ground-based remote sensing observations, specifically ALC and AERONET data, with dust information obtained from SEVIRI images (denoted above as “sat+ALC+AER”) using uniform weighting. PCNN-based reconstructions for these cases were performed using the training dataset based on ten years of CAMS reanalysis (“CAMS clim.”).

**Fig. 9:**
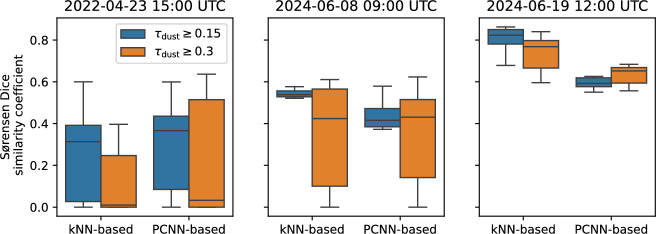
SC of kNN-based and PCNN-based reconstruction with respect to patterns from the numerical forecast ensemble for three additional test cases, 2022-04-23 15:00 UTC (left), 2024-06-08 09:00 UTC (centre), and 2022-06-19, 12:00 UTC (right). Colours indicate the threshold of $\tau _\textrm{dust}$ for the forecast to be considered as dust plume.

The SC of these additional reconstructions with respect to the operational forecast ensemble is shown in Fig. 9. When considering the 2024-06-19 case, the kNN-based reconstruction clearly performs better than the PCNN-based reconstruction. With respect to forecasts with $\tau _\textrm{dust} \ge 0.15$, the kNN-based reconstruction outperforms the PCNN-based reconstruction for the 2024-06-08 case. With respect to forecasts with $\tau _\textrm{dust} \ge 0.30$, PCNN-based and kNN-based forecasts perform on par. For 2022-04-23, the PCNN-based reconstruction performs marginally better than the kNN-based reconstruction. The high spread of calculated SC values indicates, however, relatively low agreement of the spatial patterns from operational forecasts. For comparison, the spatial agreement of individual forecast models $\tau _\textrm{dust}\ge 0.15$ for these three additional cases is shown in Fig. S8. The available models for these cases are listed in Tab. S2. Spatial patterns from the kNN-based and the PCNN-based reconstructions are shown in Fig. S9.

These additional cases indicate, that the proposed kNN-based reconstruction performs robustly. The results are at least on par with or outperforming PCNN-based reconstructions.

## Discussion

### Uncertainty

The sparsity of ground-based observations in North Africa can be overcome with PCNN-based approaches to successfully reconstruct the extent of cloud-obscured dust plumes using satellite observations only^17^. The high values of AOD which were largely obscured by clouds on 2022-03-15 presented a challenge in our original approach, resulting in one of the poorest reconstructions during the ANN’s validation stage (see Fig. 4 in^17^). In this study, we tested, how well the PCNN-based reconstruction technique performed for this dust plume over Europe. The climatological analysis of ten years of CAMS reanalysis, indicates that optically thick dust plumes ($\tau _\textrm{dust}\ge 0.30$) rarely occur north of the Pyrenees. Three different training datasets were used for performing the PCNN-based reconstructions. Notably, there was little difference in performance between the reconstruction based on the “CAMS+SEVIRI” dataset, which paired CAMS reanalysis with SEVIRI-derived cloud masks and contained $\sim 3700$ pairs and the “CAMS clim. (extremes)” dataset, which contained $\sim 2750$ pairs reanalysis fields of large dust plumes north of 36$\vphantom{0}^{\circ }$N and the associated CAMS-derived cloud masks. In case of the “CAMS+SEVIRI” dataset, no measures were taken to ensure the inclusion of a minimum number of dust plumes. The best-performing PCNN in terms of SC was trained on the “CAMS clim.” dataset, which contained $\sim 11800$ pairs of dust reanalysis fields and CAMS-derived cloud masks pertaining to moderate to large dust plumes. The improvement in reconstruction quality due to increasing the training datasets size as evaluated by the SC was small. Combined with evaluation in terms of MCC and Hausdorff distance, suggest no clear benefit of further increasing the size of the training dataset for the reconstruction quality. For comparison, our earlier study focused on North Africa required $\sim 1400$ pairs (cf.^17^).

We investigated limitations in the dust plume reconstruction approach, proposed in^17^. The PCNN-based in-painting reconstruction still added value compared to non-reconstructed dust plumes, when evaluated against different numerical forecasts as a reference (cf. Fig. 4). Thus, the PCNN-based reconstruction remains an important tool for reconstructing spatial features of cloud-obscured dust plumes and is by no means invalidated. Following suitable adaptions, the CRAI code, which underpins the PCNN-based in-painting reconstruction, has proven to be capable of restoring extreme events, as recently demonstrated for historical extreme temperature anomalies (cf.^43^). However, creating suitable training datasets for spatial dust plume reconstruction remains challenging, as the cloud geometry may influence the reconstruction quality (cf.^79^).

We proposed a simple technique, which combined ceilometer, photometer, and passive satellite observations with the help of a k-nearest neighbours classification algorithm to reconstruct partially cloud-obscured dust plumes, transported to Europe and thereby improve available information on dust plumes. This kNN-based reconstruction, alleviated the demonstrated limitations of the PCNN-based reconstruction (cf.^17^) for the dust plume during 2022-03-15 in Europe (see Figs. 5, 7, and for a direct comparison between PCNN-based and kNN-based see Fig. S7). We further found, that adding additional, more ambiguous datasets, resulted in a poorly constrained kNN-based reconstruction and, hence, in a deterioration of the reconstruction quality. We also found using directional weighting to account for atmospheric transport by wind, does not improve the reconstruction. In addition, a lack of SEVIRI observations conclusively classified as either dust-containing or dust-free due to large cloud cover poses a risk of reducing the reconstruction quality. This classification based on perceptional colour differences in the Dust RGB product is currently confined to day-time conditions. In such cases, the kNN-based method is expected to result in poor quality reconstructions, as the reconstruction would be insufficiently constrained.

Three additional dust plume cases were successfully reconstructed with both the kNN-based and the PCNN-based method and compared against the dust forecasts provided by the WMO Dust Regional Center Barcelona. The reconstructions used the combination of observational datasets, which performed best during 2022-02-15, i.e., satellite observations with AERONET and ALC data using uniform weighting, for the kNN-based reconstruction. Similarly, the PCNN trained on the CAMS reanalysis with dust plumes north of 36$\vphantom{0}^{\circ }$N with at least ten grid boxes in native CAMS resolution with $\tau _\textrm{dust}\ge 0.15$ (denoted as “CAMS clim.”) was used. Both methods reconstructed dust plumes that fall within the forecast ensemble spread. For two of these cases, the kNN-based reconstruction resulted in slightly better agreement with the operational forecast ensemble, as indicated by the median SC. In the third case, the PCNN-method performs on par with the kNN-based method. This indicates, that the kNN-based reconstruction works for different cases and can be used for forecast evaluation. Due to differences in dust occurrence inferred from different reanalysis datasets (cf.^17^), the exact plume morphology, as well as the locations of plume edges can be considered uncertain. To better encapsulate this uncertainty, we considered the individual models comprising the operational forecast ensemble provided by the WMO Dust Regional Center Barcelona as a reference (see “Machine-learning-based in-painting” section). Thus, it is not expected that the reconstructions fully reproduce the entire ensemble or even individual models of the ensemble.

Currently, both methods rely on the gray-scaling approach described in^17^, which extracts dust information from operational Dust RGB product. The colours are subject to environmental influence, such as surface emissivity, moisture content, dust plume height, or skin temperature^80^. The threshold identified in^17^ is limited to day-time conditions to reduce false positives, i.e. extracting non-dust features as dust. Refining the gray-scaling approach by using different thresholds based on the surface and/or based on moisture content or on a moisture content climatology may result in improved reconstructions for both methods. Uncertainties introduced by the gray-scaling approach affect both reconstruction methods.

### Data selection for reconstruction

We investigated the use of the dense observational networks in Europe for an improved dust plume reconstruction during large cloud coverage. We combined information from SEVIRI Dust RGB images with different (combinations of) ground-based observational datasets. All additional datasets were ambiguous and to some extent required assumptions to be made for inferring the presence of (Saharan) dust aerosol.

Ground-based remote sensing by ceilometer and photometer are especially useful for the proposed reconstruction, since they provide both an indication of elevated dust/aerosol layers and dust-free profiles. Sun photometers provide information on the mode of the aerosol size distribution, based on thresholds reported in^81,82^, it is possible to infer the presence of dust in the atmospheric column with a high degree of certainty. It is equally possible to infer the absence of dust. Ceilometer observations can provide information on the presence and absence of lofted aerosol layers beneath the cloud base. In complex situations with multiple aerosol plumes in close proximity, such as dust plumes and smoke plumes from wildfires, the different plumes may not be distinguished solely based on ceilometer observations.

**Fig. 10:**
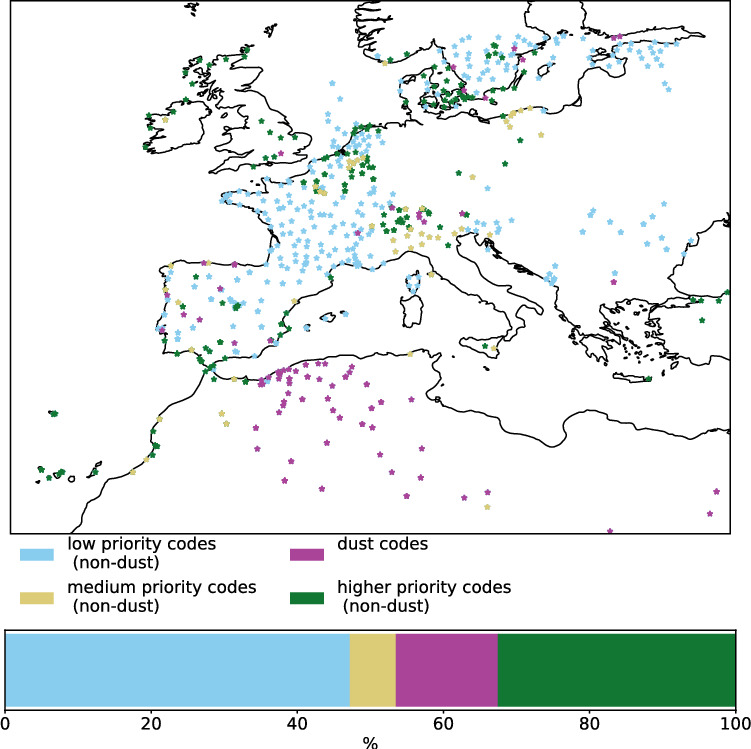
Overview of stations included in the ISD, which report data in fields MW1-MW7 on 2022-03-15 at 12 UTC. Blue denotes non-dust reports with low priority, which would have superseded by reports of dust. Yellow indicates reports, which would supersede some reports of dust, but be superseded by other reports of dust. Purple indicates reports of dust, whereas green indicates non-dust reports with higher priority, which would supersede dust reports. See Tab. S1 for the numerical values of the ISD dust codes. Note, that no station reported a thunderstorm combined with duststorm or sandstorm, which has the second-highest priority within the MW1-MW7 identifiers of the ISD. The map was created using the Python package Cartopy (version 0.24.1, https://scitools.org.uk/cartopy/docs/v0.24/index.html)^57^.

In case of observations reported in the ISD, it remained challenging to know, whether dust was observed or not, as the codes pertaining to dust are overwritten if weather deemed of a higher significance also occurs. The comparison of ISD reports during 2022-03-15 (Fig. 10) with both reanalysis and numerical forecasts (Figs. S3 and 3) further suggests that ISD reports may miss transported dust in favour of weather of less significance. Further, ISD reports may misclassify other phenomena as dust, and they do not allow distinguishing between long-range transported dust and dust stemming from local/regional emission, such as agricultural dust (cf.^83,84^). This indicates the limited use of ISD observations as constraint for the reconstruction of long-range transported dust plumes.

Saharan dust intrusions in Southern Europe can be linked to elevated levels of PM10 surface concentrations^18,20,22,26,85–89^. However, despite reducing effects stemming from industrial and traffic related aerosol sources, ambiguity remained whether elevated PM10 levels pertain to transported Saharan dust. It further remains unclear, whether the lack of elevated PM10 levels indicated a dust-free atmospheric column. Hence, in-situ PM10 observations were rendered unsuitable for this specific reconstruction task.

Ground-based remote sensing observations were found to be particularly valuable to restore the spatial extent of cloud-obscured dust plumes transported to Europe. By providing information on both the presence and absence of lofted aerosol plumes, they could further constrain the reconstruction. This underlines the potential usefulness of assimilating ALC observations, consistent with^90^. However, our study also indicated, that reconstructions of cloud-obscured Saharan dust plumes over sea surfaces remains challenging due to the lack of continuous ALC observations at sea.

## Conclusions

On 15 March 2022, an intense dust plume, which was largely obscured by clouds, was transported to Europe. The climatological analysis of dust transport to Europe and cloud co-occurrence, shows that plumes reaching as far north as the plume on 15 March 2022 are comparatively rare. Furthermore, such plumes are frequently associated with extensive cloud coverage. The dust plume was transported to Europe by an atmospheric river, which formed under favourable pressure conditions west of the North African coast despite an unfavourable large scale circulation pattern as indicated by the NAO index (see “Synoptic situation during 2022-03-15” section). The combination of these circumstances result in a challenging test case for the PCNN-based reconstruction method, developed for reconstructing dust plumes in North Africa, in Europe^17^. This test demonstrated limitations of applying the method to a region, in which dust plumes and clouds commonly co-occur. We demonstrated, that these limitations can be alleviated by a new reconstruction method using the rich observational networks across Europe. We further compare the methods for three additional dust plume cases.

We developed and tested the new kNN-based method to reconstruct information on the dust plume extent in satellite images exploiting dense observational networks in Europe. Compared to North Africa, Europe possesses a dense network of monitoring stations. The observations by these different stations are commonly ambiguous, as they do not directly pertain to dust. However, combining satellite with ground-based remote-sensing observations yielded reconstructions, which showed a higher agreement with numerical forecasts than the PCNN-based reconstructions. In other words, in regions with dense observational networks, kNN-based reconstructions can alleviate limitations of PCNN-based reconstructions.

Our investigation with kNN also indicate that more observational datasets do not necessarily add more value for dust plume reconstructions due to ambiguity in some observational data sets. Specifically, adding data for surface-level PM10 from air quality stations provided by the EEA and reports from ISD stations provided by NOAA, which have previously augmented and facilitated studies of dust events (cf.,^86–89,91–95^) , did result in poorer reconstruction quality across all evaluation metrics (see Figs. 6, S4, S5). Other data sets are more beneficial for dust plume reconstructions. One example are accurate clear-sky aerosol optical depth measurements from AERONET’s sunphotometer network. Although comparatively sparse in coverage and limited to cloud-free conditions, sunphotometer measurements at different wavelengths allow us to infer the size mode of the aerosol and gauging if aerosols are more or less absorbing. By contrast to photometer measurements, ALC observations provide height-resolved information on aerosol layers in clear and cloudy conditions, which was beneficial for the reconstructions. Ceilometer measurements provide information of lofted aerosols below clouds, which constitutes a major advantage for the reconstruction compared to in-situ PM measurements. The best reconstruction results with kNN were obtained by combining ceilometer and sunphotometer data.

The best choice of method for dust plume reconstructions depends on the degree of cloudiness and the availability of ground-based data. The kNN-based method requires ground-based remote sensing observations to constrain dust plumes against dust-free conditions. Thus, this method can be applied in regions, where such ground-based networks exist, e.g., Europe, and can reconstruct dust plumes that are obscured by clouds. The kNN-based reconstructions were performed on a single CPU (see “Reconstruction by k-nearest neighbour classification”section), i.e., is computationally inexpensive, and can be tested in other regions of interest with suitable observational networks. Unlike the PCNN-based method, the kNN-based reconstruction does not require long-term data for training, as only information available at the time of interest is used. The PCNN-based reconstruction method requires only reanalysis fields of dust aerosol optical depth and temporally corresponding cloud masks. Therefore, this method is especially suited for reconstructions in regions with sparse ground-based remote sensing observations and works best in clear to moderately cloudy conditions, e.g., the Sahara desert in North Africa. Training for this method is computationally more expensive compared to kNN (here done on GPUs, see “Machine-learning-based image in-painting” section), but once the training has been completed the reconstruction of additional cases is fast. Both methods including their training are associated with a substantially lower computational burden than performing dust forecasts with traditional prediction models and obtain dust plumes that fall within the forecast ensemble spread.

## Methods

### Satellite-borne observations

To infer the presence and absence of dust in cloud-free conditions from SEVIRI observations, we relied on the Dust RGB images. These false colour images which show lofted dust plumes in bright magenta and were provided by EUMETSAT^59^. RGB images are comprised of a red (R), green (G), and blue (B) channels. All colours can be decomposed to RGB values by assigning a value between 0 and 255 to the respective channels. For example, red can be decomposed into $RGB = (255,0,0)$ and yellow to $RGB = (255,255,0)$. The Dust RGB images are created by assigning different combinations of spectral measurements of brightness temperatures by the SEVIRI instrument in channels centred around $\lambda =8.7\,\mu$m, $\lambda =10.8\,\mu$m, and $\lambda =12.0\,\mu$m to the RGB channels^58^.

Sand surfaces, which contain quartz mineral, are displayed in light blue, while clouds appear in red-brownish colours, dark green and/or black^58,80^. Magenta colours for dust plumes mainly arise from differences in spectral emissivity at the different wavelength bands. Large emissivity of small quartz mineral particles ($0-45\,\mu$m) at $\lambda = 8.7\,\mu$m results in a small brightness temperature difference between $\lambda = 8.7\,\mu$m and $\lambda = 10.8\,\mu$m. The brightness temperature between these two wavelengths modulates the RGB composite’s green channel, with low values in the green channel giving rise to magenta shades^58,80^.

The PCNN-based in-painting algorithm (cf. “Machine-learning-based image in-painting” section) required quadratic images as input. Here, we use images with 128 by 128 pixels, which cover the region bound by latitudes of 24$\vphantom{0}^{\circ }$N and 60$\vphantom{0}^{\circ }$N, and the longitudes of 20$\vphantom{0}^{\circ }$W and 52$\vphantom{0}^{\circ }$E. This resulted in an individual pixel spanning 0.28125$\vphantom{0}^{\circ }$ in North-South direction and 0.5625$\vphantom{0}^{\circ }$ in East-West-direction.

Banks et al.^80^ investigated the effect of environmental conditions, such as atmospheric water vapour content, surface skin temperature, and height of the dust plume on the colour in MSG-SEVIRI Dust RGB images over North Africa. Following the the gray-scaling approach in^17^, for which Dust RGB images are converted to a Cartesian colour space, the non-obstructed dust plume information is extracted using perceptional colour differences^96^ with respect to magenta ($RGB = (255,0,255)$). We assigned white to magenta pixels ($RGB = (255,0,255)$) and grey values based on the calculated perceptional colour differences to magenta. All pixels exceeding a perceptional colour difference of $\Delta E = 51.9$ were considered to not contain dust information and were, thus, assigned black. Information on dust- and cloud-free conditions was obtained analogously from Dust RGB images, but by considering all pixels with a perceptional colour difference of $\Delta E \le 66.7$ from cyan ($RGB = (0,255,255)$) as dust- and cloud-free. This value was based on^80^.

The PCNN-based dust plume reconstruction method required for both the training and the actual reconstruction a cloud mask. We employed the operational SEVIRI cloud mask product (CLM), which was provided by EUMETSAT^97^. The CLM data covered the same spatial extent and has the same spatial resolution as the Dust RGB data used. For both the Dust RGB images and the CLM product, the reduced resolution data was provided by EUMETSAT’s application programming interface. SEVIRI observations were provided with a temporal resolution of 15 minutes.

We investigated the effect of considering atmospheric motion on the reconstructions, for which we use atmospheric motion vectors (AMVs), derived from SEVIRI observations^70^ in addition to reanalysis data (see Fig. 8). AMVs are derived by tracking clouds or water vapour features in consecutive geostationary satellite images. For this approach specific cloud or in cloud-free conditions water vapour features are identified, tracked and assigned to a specific height^73^. EUMETSAT provides estimates of the associated height by applying the cross-correlation contribution method^74^. To obtain a horizontal coverage of reported AMVs, which is as high as possible, as a basis for interpolating the horizontal motion information onto a regular grid, we do not consider the height estimation of the associated AMVs. Hence, we may potentially combine information on atmospheric motion with information on elevated dust plumes at different heights. Since no features may be available to track for the determination of the AMV product, it is typically reported on an irregular grid. We interpolated the AMV product to the same resolution as the Dust RGB, using linear interpolation with standard functions implemented in the package SciPy (version 1.16.1)^98^.

### Ground-based observations

Height resolved information of the aerosol backscattering coefficient and the cloud height is operationally observed by automated lidar and ceilometers (ALC). The ALC observational data is collected and provided by the E-PROFILE observation programme, which is coordinated by the European Meteorological Network (EUMETNET)^50^. The aerosol backscatter coefficient as reported within E-PROFILE can pertain to any type of atmospheric aerosol. Individual dust outbreaks have been studied using ALC observations provided by E-PROFILE^88,90,99^. Further, ALC observations are used for a routine early detection of lofted aerosol layers in Romania^100^. We identified lofted aerosol layers by the attenuated backscatter $\beta _\textrm{att}$. If the profile of the attenuated backscatter below the clouds exceeded values of $\beta _\textrm{att}=2.0\,$Mm$\vphantom{0}^{-1}$sr$\vphantom{0}^{-1}$, we identified a lofted aerosol layer. This threshold on ceilometer observations was based on reports of a Saharan dust outbreak over the Iberian Peninsula^90^. In clear air, $\beta _\textrm{att}$ takes values of $\beta _\textrm{att} \approx 0\,$Mm$\vphantom{0}^{-1}$sr$\vphantom{0}^{-1}$, whereas for dust plumes over the Iberian Peninsula values to up to $\beta _\textrm{att} = 15\,$Mm$\vphantom{0}^{-1}$sr$\vphantom{0}^{-1}$ have been reported^90^. To reduce the risk of misidentifying plumes attributable to local effects, we considered only plumes with a height of at least $750\,$m above ground. Multi-wavelength observations, including observations of the lidar ratio and the linear depolarisation ratio, which allow for more accurate aerosol typing (cf.,^101^), reported by the European Aerosol Research Lidar Network (EARLINET), observed dust plume heights in March 2022 above 1 km^19^. Thus, we considered the risk of falsely excluding dust aerosol layers to be negligible. We further performed a basic noise removal, by considering only aerosol layers, if the values of $\beta _\textrm{att}$ height bin directly beneath or above the aerosol layer were $\beta _\textrm{att}>0.0$. As an additional quality check, we considered only observations, which were flagged as having assured signal quality. The ALC network provides measurements every five minutes and consists mainly of Jenoptik CHM15k instruments and Vaisala CL 51 and CL 31 instruments^50,102^. The CHM15k operates at a wavelength of 1064 nm, while the CL 31 and CL 61 instruments operate at a wavelength of 910 nm. In total, the E-PROFILE observation programme incorporates 446 ALC stations, of which 280 in 16 different countries in Western, Southern, and Central Europe were considered. After noise filtering and accounting for gaps in the observational data, 134 stations remained in our dataset.

Ground-based photometers within the Aerosol Robotic Network (AERONET), which is coordinated by the US National Aeronautics and Space Administration (NASA), provided another data source. By passively observing direct and indirect radiation, photometers provide information on the columnar aerosol optical thickness and the Ångström exponent å$\vphantom{0}_{\lambda _1, \lambda _2}$. However, these measurements require cloud-free conditions along the path between the instrument and the sun^51,69^. AERONET observations have been used previously to study dust outbreaks over the Iberian Peninsula^81^. Photometer observations of absorbing ($\tau >0.15$) coarse mode aerosol (å$\vphantom{0}_\mathrm {440 - 870 nm}<0.75$) are classified as mineral dust dominated observations, following the thresholds given by Basart et al.^81^. Measurements reported by Kumar et al.^82^ further support these thresholds, especially when distinguishing between absorbing mineral dust and weakly or even non-absorbing sea salt/marine aerosol. Here, we used AERONET level 1.5 data. Of 126 stations operating during March 2022, 19 stations reported the AOD on 2022-03-15 at 12 UTC. The sampling rate depended on the site, but was typically between two and six minutes. In March 2022, the highest value of $\tau$ reported via AERONET was $\tau = 3.039$. This value was associated with coarse mode aerosol.

We also considered reported weather codes reported in the Integrated Surface Database (ISD) maintained and provided by NOAA’s National Center for Environment Information (NCEI)^52^. The ISD combines human and automated observations of meteorological data from different stations. Present and past weather codes are reported hierarchically. Specifically, weather events are encoded with a numerical two-digit value, an event with encoded with a higher numerical valued is deemed more significant and supersedes events encoded with a lower value. E.g., weather phenomena such as fog and/or precipitation may supersede dust-related weather codes and, thus, overwrite the reported values, while dust-related weather events may overwrite fog and/or precipitation events during the preceding hour and changes in cloud cover (cf.^103^). An overview over all ISD codes pertaining to dust, including the description and the respective field identifier, is given in Tab. S1. In the Lake Eyre Basin, an Australian dust source region, the supersession of dust events due to this hierarchical reporting led to data loss, resulting in around 7% of all dust days and 15% of dust storm days per year not being reported^104^. Thus, no reporting of dust, does not necessarily indicate the absence of dust. Weather station data has been used in the past to study dust events and their climatology in Europe, North Africa and world-wide^91–94^. Fig. 10 shows the spatial distribution of dust and non-dust reports of present weather on 2022-03-15, 12 UTC as indicated by the ISD field identifiers MW1-MW7 (see Tab. S1). Colours indicate the priority of reports, which are classified according to whether they are superseded by all (blue), some (yellow) reports of dust (purple), or whether they supersede dust reports (green).

**Fig. 11:**
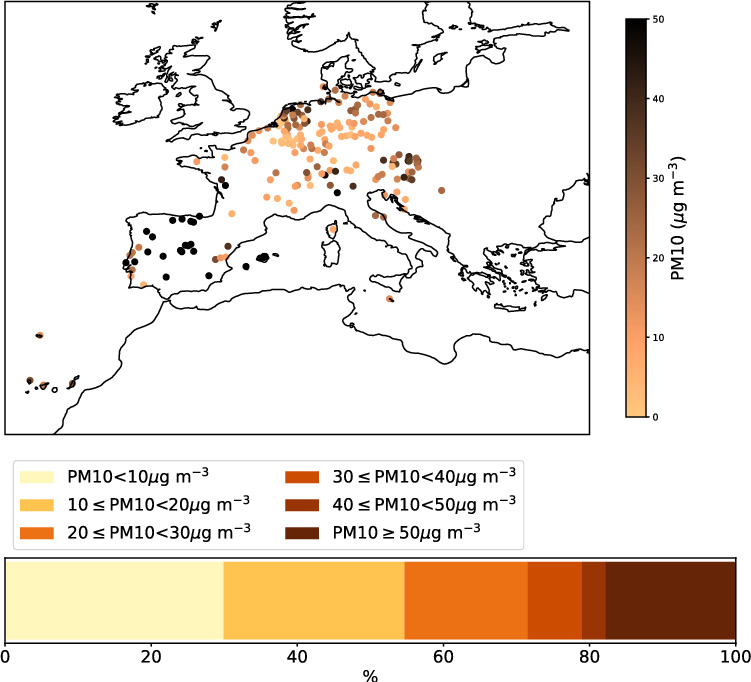
Overview over reported PM10 measurements on 2022-03-15 at 12 UTC, provided by EEA rural background air quality stations. The map shows the reported PM10 values, the colour bar indicates the fraction of PM10 reports within bins of $10\,\mu$g m${-3}$. The map were created using the Python package Cartopy (version 0.24.1, https://scitools.org.uk/cartopy/docs/v0.24/index.html)^57^.

As a last data source, we considered in-situ observations of large particulate matter (PM10), which were provided by the European Environment Agency (EEA). PM10 refers to particles with an aerodynamic diameter smaller than $10\,\mu$m^22^. Saharan dust events are known to increase PM10 levels in Southern Europe^20,26,86–89^. PM10 data only pertains to observations at the surface and does not distinguish between aerosol types^22,85^. Air quality station operated in accordance with EU air quality legislation can be grouped according to the classification of the area, in which they are positioned, i.e. rural, suburban, and urban. They can further be classified according to their type, whether they are traffic, industrial or background sites^22^. To reduce the impact of local air quality effects due to heavy traffic or industrial emissions, we considered only rural background sites. Rural background sites, as defined by EU air quality legislation, are required to be at least 20 km away from urban and industrial sites. Further, they are required to be away from local emission sources and locations which are prone to the formation of inversions close to the ground^21^. We considered rural background stations reporting an hourly average PM10 concentration of at least $50\,\mu$g m$\vphantom{0}^{-3}$ as an intense dust episode. For Austria, Germany, France, Italy, and Poland, annual average PM10 concentrations of $12.8 - 21.6\,\mu$g m$\vphantom{0}^{-3}$ were reported during 2017^22^. For dust outbreaks in the Mediterranean, moderate to intense events, defined as $30.0 - 99.0\,\mu$g m$\vphantom{0}^{-3}$ PM10 dust account for 5 - 25% of African dust days during the period between 2001-2010. Low intensity episodes (PM10 of $1.0 - 10.0\,\mu$g m$\vphantom{0}^{-3}$) account for 50-70% of African dust days^87^. While our chosen threshold of $50.0\,\mu$g m$\vphantom{0}^{-3}$ may not capture all dust events, dust events are frequently exceeding this threshold^86,95^. However, while only considering rural background stations reduced the risk of wrongly classifying emissions due to heavy traffic or industrial processes, it did not safeguard against other misclassifications. An overview over the reported PM10 concentrations at rural background stations on 2022-03-15 at 12 UTC is shown in Fig. 11.

### Reanalysis and forecast fields

In addition to satellite products, our study used reanalysis data of the dust aerosol optical depth and the total cloud cover as provided by the CAMS reanalysis^55,56^. We also used the reanalysis of the dust aerosol mixing ratios. CAMS reanalysis is provided at the main and intermediate synoptic times, i.e., in three-hourly intervals starting each day at 00:00 UTC. To assess the synoptic situation, as well as, for tests of the data-driven reconstruction, we used meteorological data from ERA5 reanalysis fields^72,105^. Specifically, we used the horizontal wind, the specific humidity, mean sea level pressure, geopotential height, cloud fraction, and total cloud cover.

Reanalysis products are commonly used as a reference. However, dust plume patterns between CAMS reanalysis and the Modern-Era Retrospective analysis for Research and Application, Version 2 (MERRA-2) reanalysis^106^ in North Africa differ^17^. To check the reconstructions’ robustness against deviations in the reference dataset the evaluation is performed against an ensemble of numerical dust forecasts of dust AOD. The numerical forecasts were provided by the World Meteorological Organisation (WMO) Barcelona Dust Regional Center and the partners of the Sand and Dust Storm Warning Advisory and Assessment System (SDS-WAS) for Northern Africa, the Middle East, and Europe. The individual models, including domain and native horizontal resolution, as well as the issuing time, are listed in Tab. 1. Forecasts are provided at the main and intermediate synoptic times, i.e. every three hours starting from 00:00 UTC. All forecasts provided within this framework cover a geographical area, which is bound by the latitudes of 0$\vphantom{0}^{\circ }$N and 65$\vphantom{0}^{\circ }$N, as well as the longitudes of 25$\vphantom{0}^{\circ }$W and 60$\vphantom{0}^{\circ }$E. During 2022-03-15 twelve operational forecasts, as well as, the multi-model median forecast were available. When not considering the entire forecast ensemble, we focused on the forecasts on three forecasts, CAMS-IFS, MONARCH, and the ensemble median, MULTI-MODEL. CAMS-IFS assimilates aerosol information explicitly and uses the model underpinning the CAMS reanalysis. MONARCH is the WMO Barcelona Dust Regional Center’s reference model. The ensemble median, which is operationally provided by the WMO Barcelona Dust Regional Center, can be considered as a very robust indicator of dust presence. To calculate the multi-model median forecast across the entire available model ensemble, the forecasts are re-gridded to a horizontal resolution of $0.5^{\circ }\times 0.5^{\circ }$.

**Table 1:** Overview of output from numerical forecast models provided by the WMO Barcelona Dust Regional Center and the partners of the Sand and Dust Storm Warning Advisory and Assessment System (SDS-WAS) for Northern Africa, the Middle East and Europe. Model names are as indicated by WMO Barcelona Dust Regional Center. MULTI-MODEL denotes the median forecast as provided by the WMO Barcelona Dust Regional Center. * indicates model not available during 2022-03-15, but during at least one of the additional cases considered.

Model	Domain	Native horizontal resolution	Issuing time	References
ALADIN	Regional	$25\,\textrm{km}\times 25\,\textrm{km}$	00 UTC	^107,108^
BSC-DREAM8b	Regional	$\frac{1}{3}^{\circ }\times \frac{1}{3}^{\circ }$	12 UTC	^109–111^
CAMS-IFS	Global	$\sim 9\,\textrm{km}$ $\vphantom{0}^{a}$	00 UTC	^75^
DREAM8-CAMS	Regional	$\frac{1}{3}^{\circ }\times \frac{1}{3}^{\circ }$	00 UTC	^112,113^
EMA-RegCM4	Regional	$45\,\textrm{km}\times 45\,\textrm{km}$	00 UTC	^114^
LOTOS-EUROS	Regional	$0.5^{\circ }\times 0.25^{\circ }$	00 UTC	^115^
MOCAGE*	Global	$1^{\circ }\times 1^{\circ }$	00 UTC	^116^
MONARCH	Regional	$\frac{1}{3}^{\circ }\times \frac{1}{3}^{\circ }$	12 UTC	^77,78^
NASA-GEOS	Global	$0.25^{\circ }\times 0.3125^{\circ }$	00 UTC	^117^
NCEP-GEFS	Global	$1^{\circ }\times 1^{\circ }$	00 UTC	^118^
NOA	Regional	$0.19^{\circ }\times 0.22^{\circ }$	12 UTC	^119^
SILAM	Global	$0.5^{\circ }\times 0.5^{\circ }$	00 UTC	^120^
WRF-NEMO	Regional	$18\,\textrm{km}\times 18\,\textrm{km}$	00 UTC	^121^
ZAMG-WRF-CHEM*	Regional	$0.2^{\circ }\times 0.2^{\circ }$	00 UTC	^122^
MULTI-MODEL (median)	–	$0.5^{\circ }\times 0.5^{\circ }$	12 UTC	^16,67^

$\vphantom{0}^{a}$CAMS-IFS uses a octahedral reduced Gaussian grid (O1280) with a horizontal distance of $8-10\,\textrm{km}$ between grid points^67^

The dust forecasts were re-gridded bilinearly to the same region of interest and spatial resolution as the SEVIRI Dust RGB images with the software package Climate Data Operators (CDO, version 2.0.6.,^123^).

### Reconstruction methods

**Fig. 12:**
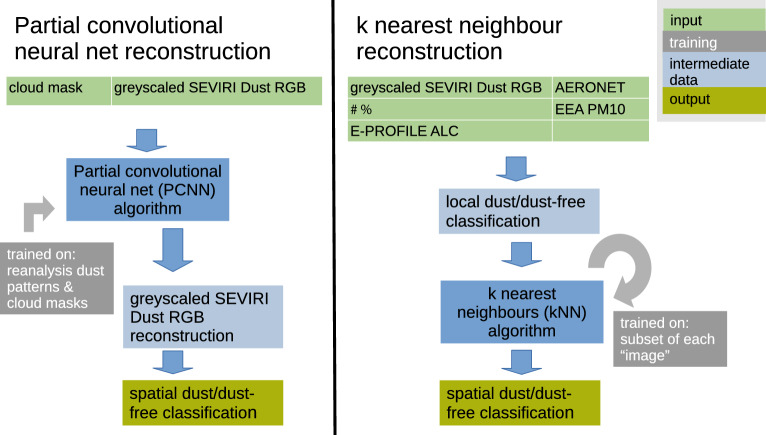
Schematic representation of the two reconstruction methods, the training approach, and the required input data.

The reconstruction methods are illustrated in Fig. 12. Both methods essentially perform a classification task to identify each pixel/grid box as dust-containing or dust-free. The PCNN-based reconstruction leverages patterns of dust plumes and cloud masks included in the training dataset. Training is computational expensive, but the reconstruction of cloud-masked, greyscaled SEVIRI Dust RGB images itself is computationally cheap (cf.^17^). These reconstructed greyscaled images are then converted into the binary dust-containing/dust-free classification. For more detailed information, please see “Machine-learning-based image in-painting” section. For the kNN-based reconstruction, SEVIRI Dust RGB images are paired with (combinations of) other observations. These observations are individually classified into dust-containing and dust-free (the assumptions are described in “Reconstruction by k-nearest neighbour classification” section). The pixels/grid boxes are then classified using the kNN-approach. The optimal value of *k*, the number of nearest neighbours to consider, is determined for each combination individually, by splitting the available data into a training and a test dataset.

#### Machine-learning-based image in-painting

We employed the climatereconstructionAI algorithm^42,60^ trained on cloud-masked CAMS dust reanalysis fields to restore the spatial extent of cloud-obscured dust plumes as previously proposed in^17^. To do so, we created three different versions by employing three different training datasets:

1. “CAMS+SEVIRI”, obtained by combining CAMS dust AOD reanalysis fields with temporally matched SEVIRI CLM-derived cloud masks from 2020-09-01 to 2021-12-31 as proposed in^17^,
2. “CAMS clim.”, obtained by combining CAMS dust AOD reanalysis fields with corresponding CAMS-derived cloud masks for dust moderate to severe European dust events (i.e., at least 10 grid boxes in native CAMS resolution north of 36$\vphantom{0}^{\circ }$ with $\tau _\textrm{dust}>0.3$) between 2010-01-01 and 2019-12-31,
3. “CAMS clim. (extremes)”, obtained by combining CAMS dust AOD reanalysis fields with corresponding CAMS-derived cloud masks for dust severe European dust events (i.e., at least 100 grid boxes in native CAMS resolution north of 36$\vphantom{0}^{\circ }$ with $\tau _\textrm{dust}>0.3$) between 2010-01-01 and 2019-12-31.

The training dataset, denoted as “CAMS+SEVIRI”, follows the approach in^17^. Hence, this dataset was not constructed to contain a minimum number of dust plumes transported to Europe. To extend the temporal coverage and to explicitly include dust plumes transported to Europe, the training datasets “CAMS clim.” and “CAMS clim. (extremes)” were created. Cloud masks were obtained from CAMS reanalysis by rounding the total cloud cover, which is reported as a number between 0 and 1, to the nearest integer. The resulting training datasets consist of 3681, 11760, and 2749 pairs of CAMS reanalysis fields and cloud masks, respectively. Potential differences between the geometrical features of the cloud masks derived from SEVIRI observations and CAMS reanalysis may affect the quality of the reconstruction (cf.^79^). Training was performed on resources provided by the German Climate Computing Center (Deutsches Klimarechenzentrum, DKRZ). Each computational node of the used partition consists of two CPUs (AMD 7713) and four NVIDIA A100 GPUs.

#### Reconstruction by k-nearest neighbour classification

We combined data from satellite observations and ground-based observations to estimate the horizontal extent of the dust plume by using the k-nearest neighbour method (kNN)^53,54^. The kNN method was previously used to spatially extend observation or model data (cf.^124^), and statistical downscaling of numerical weather prediction models^125^.

Specifically, we mainly used the kNN method as implemented in the Python package scikit-learn (version 1.7.2)^126^. For the dust plume reconstruction, two classes were considered: dust-free and dust-containing. The spatial extent of the dust plume was reconstructed by classifying each grid point accordingly. The classification of the initial datasets depended on the respective underlying subset of data, as discussed above.

Using scikit-learn standard functions, the dataset was divided into a training dataset and a test dataset. The test data set consisted of 25% of the original data set. Points in the test dataset with a different classification than all points of the training data set within a distance of 75 km were exchanged with a point from the training dataset with the same indicator. The distance between the points is calculated using Vincenty’s formulae for the inverse problem, as given by Karney^127^. These formulae allowed us to obtain the shortest path between two points based on their geographical coordinates, assuming the Earth to be an ellipsoid.

Training was performed weighing the points uniformly and with an inverse of the distance, so that closer points have a larger impact. Prior to reconstructing the dust plume, for each combination of observational datasets and each weight, the optimal value of *k* is determined. To do so, we determined the accuracy of the classification by classifying the points of the test dataset according to their location. Starting from $k=2$, *k* was increased by 1 until $k=76$. We chose the value of *k* with the highest accuracy, i.e. with the highest number of correctly classified locations for the reconstruction at hand. This search for the optimal value of *k* is performed for every individual classification.

By weighing points simply with the inverse distance, we potentially do not take into account the specifics of dust transported to Europe. We accounted for atmospheric motion by implementing a custom kNN-algorithm. This custom algorithm did not use the Euclidean distance *d*, but uses another distances metric, the semi-latus rectum *p* of an ellipse with a focal point around the point of interest. The semi-latus rectum is calculated as (cf.^128^):

1 $$\begin{aligned} p = d (1 - e \cdot \cos \alpha ) \end{aligned}$$

The ellipse’s eccentricity is denoted by *e*. For this study, a constant value of $e = 0.75$ was chosen. Here, $\alpha$ denotes the wind direction. In other words, the ellipse is rotated, so that values, which lie in the direction, from which dust is expected to be advected, are weighted higher. $\alpha$ is obtained in two different ways. Firstly, by using an interpolation of SEVIRI’s atmospheric motion vector (AMV) product, which may not refer to the atmospheric layer containing the majority of dust, but to layers containing the cloud top or in cloud-free cases containing water vapour features. Secondly, by using fields of the horizontal wind vectors obtained from ERA5 reanalysis at different pressure levels between 600 and 850 hPa. CAMS reanalysis indicated a high dust concentration at these pressure levels in Europe.

Reconstructions with the kNN-method were performed on a single Intel i5-1235U CPU.

**Fig. 13:**
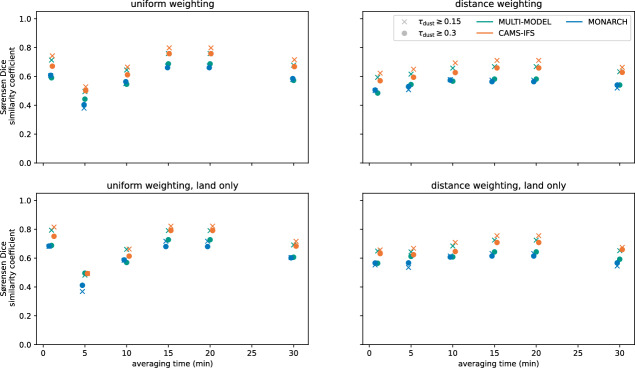
SC between reconstructions, which use SEVIRI observations and ground-based remote sensing observations, and numerical dust plume forecasts from three models as a function of averaging time of the attenuated backscatter profiles reported from ceilometer measurements (ALC). The left column used a kNN classification with uniform weighting and the right column with distance weighting. The top row considered dust plumes in the entire area of interest, whereas, the bottom row only shows the SC for dust plumes over land areas. Symbols and colours are as in Fig. 4.

Profiles of the attenuated backscatter coefficient as measured by ceilometers were reported with a high temporal resolution of 5 minutes. In addition to the above-mentioned simple noise filtering approach, averaging was performed by calculating the arithmetic mean of all reported profiles during the averaging period leading up to the time of interest, i.e. if the averaging time was 10 minutes, we considered all reported profiles within the ten minutes prior to the time of interest. We tested averaging times of 5, 10, 15, 20, and 30 minutes, as well as no temporal averaging. No temporal averaging is indicated by an averaging time of 0 minutes. Testing was performed by using satellite-derived dust plume information with averaged ALC profiles and AERONET data to perform a kNN-classification. The classifications are compared to numerical forecasts, as done when evaluating machine-learning based reconstructions. For both weighing the stations uniformly and based on their distance, choosing averaging periods of 15 and 20 minutes result in the highest values of the Sørensen-Dice similarity coefficient, as can be seen in Fig. 13. As an averaging time of 15 minutes coincides with the temporal resolution of the SEVIRI observations, we chose 15 minutes.

### Evaluation metrics

To evaluate the reconstructions, we used three different metrics, the Sørensen-Dice similarity coefficient *SC*, the Matthews correlation coefficient *MCC*, and the Hausdorff distance.

The overlapping between two sets *A* and *B* is quantified by the Sørensen-Dice similarity coefficient^61,62^. The SC between two sets *A* and *B* is defined as:

2 $$\begin{aligned} SC=2 \frac{|A\cap B|}{|A| + |B|} \end{aligned}$$

The cardinality of a set $*$, i.e. its number of elements, is denoted by $|*|$. *SC* takes values between 0 and 1, with 0 indicating no overlap between the two sets *A* and *B* and 1 indicating perfect overlap. When considering one of the sets as reference, the SC penalises all deviations, regardless of the spatial distance to the reference.

The Matthews Correlation Coefficient (MCC) has been both used for binary and multiclass classifications, although it is particularly useful for binary classification tasks^63^. It can be calculated from the entries of the standard confusion matrix, which contains the number of true positives (TP), false positives (FP), true negatives (TN), and false negatives (FN)^64^:

3 $$\begin{aligned} MCC = \frac{TP \cdot TN - FP \cdot FN}{\sqrt{(TP + FP)\cdot (TP + FN )\cdot (TN+FP)\cdot (TN+FN)}} \end{aligned}$$

The MCC takes values between +1 and -1. Values of +1 indicate a perfect prediction, whereas, values of -1 indicates a perfectly wrong prediction, i.e., all positives are classified as negatives and vice versa. Values of $MCC \approx 0$ indicate a prediction similar to random guessing. To achieve high values of the MCC the majority of both positive and negative cases has to be classified correctly. However, as can be inferred from Eq. 3, the MCC does not account for “narrow” misses. As for the SC, misclassifications are penalised by the MCC regardless of the spatial proximity between the wrongly classified pixel and the respective correctly valued pixel.

Image (dis)similarity can be also quantified using the Hausdorff distance^65,66^. The directed Hausdorff distance *d*(*A*, *B*) is the largest distance of a point in the test image *A* to any point in the reference image *B*. However, the directed Hausdorff distance is usually asymmetric, i.e. $d(A,B)\ne d(B,A)$. The Hausdorff distance or symmetric Hausdorff distance *D* is then defined as^65,66^

4 $$\begin{aligned} D = \max {\left( d(A,B), d(B,A)\right) }. \end{aligned}$$

Unlike the other two evaluation metrics, the Hausdorff distance is robust to small deviations^65^. The directed Hausdorff distance was used in^17^ to evaluate dust plume reconstructions. We calculated the directed Hausdorff distance using its SciPy implementation^98^, which is based on^66^.

## Synoptic situation during 2022-03-15

**Fig. 14:**
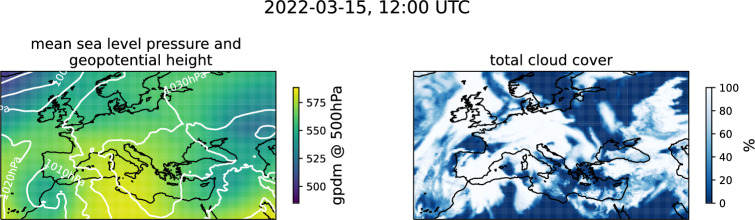
Overview of synoptics situation on 2022-03-15 at 12:00 UTC showing mean sea level pressure and geopotential height at the 500 hPa pressure surface (left), and the total cloud cover (right). Maps were created using the Python package Cartopy (version 0.24.1, https://scitools.org.uk/cartopy/docs/v0.24/index.html)^57^.

To provide additional context, a brief analysis of the synoptic situation on 2022-03-15 at 12 UTC was performed. This analysis used ERA5^71,72^ and CAMS^55,56^ reanalysis data. The synoptic situation was characterised by a low pressure area over Morocco, the extra-tropical cyclone Celia (cf.^18^), as can be seen in fields of the mean sea level pressure and the geopotential height at the 500 hPa pressure level (Fig. 14, left panel). The surface low pressure area began to form on 2022-03-13 over the Atlantic west of the Iberian Peninsula. During the course of 2022-03-14, the surface low pressure area deepened and reached Morocco. These conditions of a cyclone to the west and a ridge to the east and south favoured the formation of atmospheric rivers (cf.^28^). To complement this overview, the right panel of Fig. 14 shows the total cloud cover from reanalysis, which indicates the large area, in which dust may potentially be obscured.

This atmospheric river is included in the global atmospheric river catalog (version 4.0,^129^), which is based on ERA5 reanalysis and provides an overview over atmospheric rivers between 1940 and 2023 at six-hourly temporal and $0.25^{\circ }$ spatial resolution. The atmospheric rivers in this overview were identified using the Tracking Atmospheric Rivers Globally as Elongated Targets (tARget) algorithm^27,130,131^. In this database atmospheric rivers are identified by the integrated water vapour transport (IVT) and the shape of the detected IVT feature^130^. The IVT can be calculated by^4,130^:

5 $$\begin{aligned} IVT = \sqrt{\left( \frac{1}{g} \int _{1000\,\text {hPa}}^{300\,\text {hPa}} qu \textrm{d}p\right) ^2+\left( \frac{1}{g} \int _{1000\,\text {hPa}}^{300\,\text {hPa}} qv \textrm{d}p\right) ^2} \end{aligned}$$

Here, *u* and *v* denote the horizontal and meridional component of the horizontal wind. The specific humidity is indicated by *q* and the gravitational acceleration is $g=9.80665\,$m s$\vphantom{0}^{-2}$. In addition to the shape criterion, the IVT has to exceed the location- and season dependent 85th percentile, but at least $\textrm{IVT} = 100\,$kg m$\vphantom{0}^{-1}\,$s$\vphantom{0}^{-1}$^130^, for an event to be classified as atmospheric river.

**Fig. 15:**
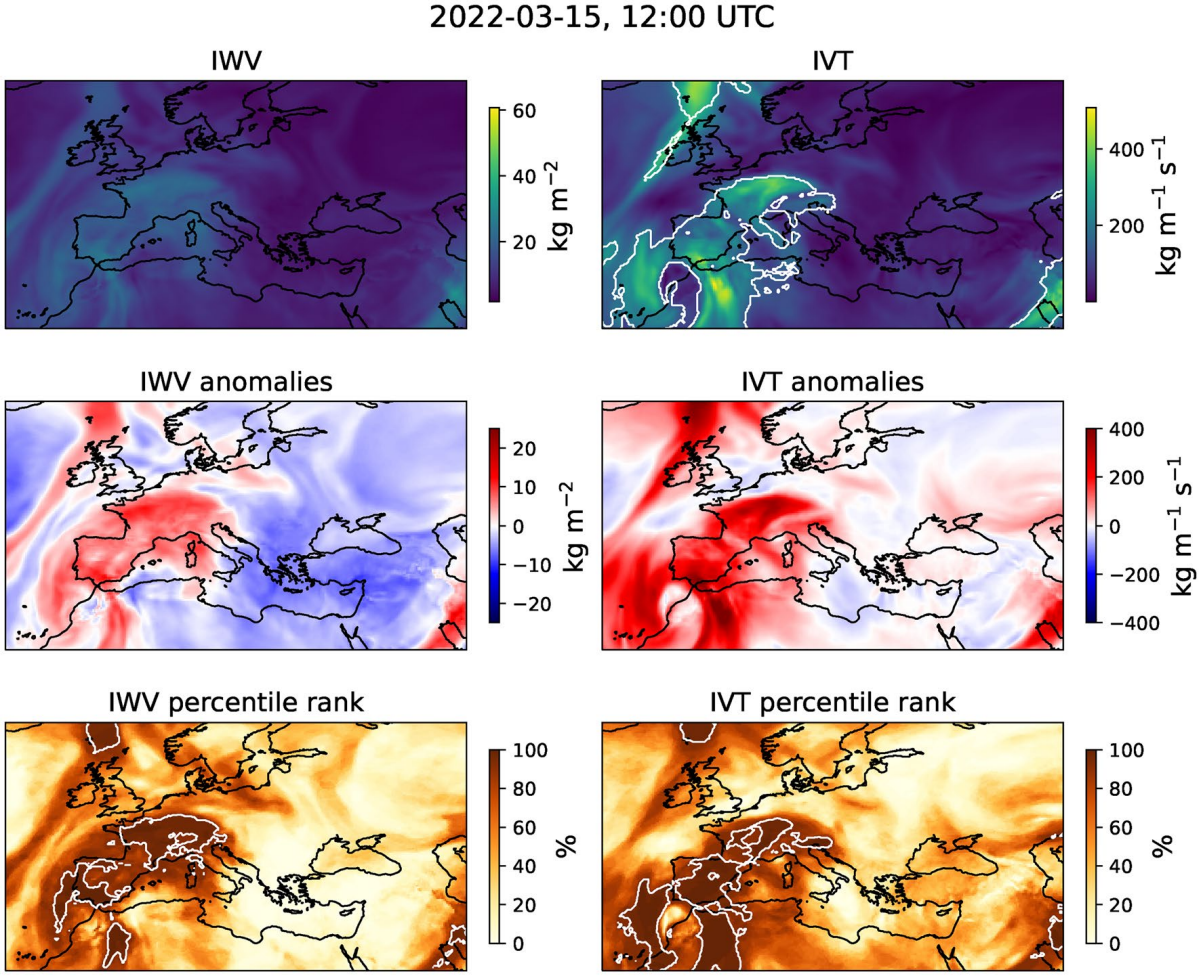
Atmospheric river diagnostics, specifically the integrated water vapour (IWV, left column) and integrated water vapour transport (IVT, right column) on 2022-03-15 at 12:00 UTC as determined from ERA5 reanalysis. The top row shows the values, whereas, the middle row shows the respective anomalies with respect to the average values on 15 March, 12:00 UTC during 1980 until 2020. Solid lines in the top right panel indicate areas, which exceed the IVT threshold proposed by^130^. The bottom row shows the percentile rank of the IWV and the IVT, i.e. the fraction of values during the period between 1980 and 2020 which are below the values during 2022-03-15 at 12:00 UTC. Solid lines mark 95%. Maps were created using the Python package Cartopy (version 0.24.1, https://scitools.org.uk/cartopy/docs/v0.24/index.html)^57^.

The atmospheric river during 2022-03-15, 12 UTC was associated with an unusually high moisture content and transport, as indicated by the integrated water vapour (IWV, Fig. 15, left) and the integrated water vapour transport (IVT, Fig. 15, right). Solid lines on the top right represent the IVT threshold for an event to be classified as atmospheric river as proposed in^130^. The absolute values are shown in the top row, whereas, the middle row indicates the anomaly with respect to the mean values for 15 March at 12:00 UTC from 1980 until 2020. The Iberian Peninsula, France, southern Germany, Switzerland and northern Italy were characterised by high positive anomalies of both the IWV and the IVT on 2022-03-15. In addition, we provide the respective percentile rank of the values during 2022-03-15 with respect to the period from 1980 until 2020. The percentile rank (cf.^132^) is the fraction of values during the reference period below the values during 2022-03-15. Solid lines here indicate a percentile rank of 95%. The high positive anomalies of IWV and IVT coincide with a high percentile rank. This high moisture content lead to the widespread cloud cover.

Further, this atmospheric river was unusual with respect to the large scale circulation pattern. The probability of dust transport towards the Iberian Peninsula increases with increasing NAO index, but the occurrence of atmospheric rivers decreases^2,4^. The daily NAO index, provided operationally by the United States National Oceanic and Atmospheric Administration (NOAA),^133^ on 2022-03-15 took a value of 1.933. This is the largest value for the 15th of March since 1950 and the ninth-largest value during March^134^. The daily NAO index value was calculated by NOAA from empirical orthogonal functions of the geopotential height on the $500\,$hPa pressure level^135^. The location of the cyclone and the ridge tended to favour the formation of atmospheric rivers, whereas, the large scale patterns as expressed by the NAO index tend to suppress the formation of atmospheric rivers.

## Supplementary Information

Supplementary file 1.

## Data Availability

The automated lidar and ceilometer (ALC) data set can be obtained from the Comprehensive Environmental Data Archive (CEDA) via https://data.ceda.ac.uk/badc/eprofile/data. Sun photometer data can be obtained via NASA’s AERONET data base via https://aeronet.gsfc.nasa.gov/. The SEVIRI RGB composite images (collection ID: EO:EUM:DAT:MSG:DUST), SEVIRI cloud masks (collection ID: EO:EUM:DAT:MSG:CLM), and SEVIRI Atmospheric Motion Vectors (collection ID: EO:EUM:DAT:MSG:AMV) can be accessed using EUMETSAT’s data store, https://data.eumetsat.int. Access to PM10 data is provided by the European Environment Agency via https://eeadmz1-downloads-webapp.azurewebsites.net/. ERA5 and CAMS reanalysis products are provided by the Copernicus Atmospheric Monitoring Service. CAMS reanalysis can be obtained from https://ads.atmosphere.copernicus.eu/#!/home. ERA5 reanalysis can be obtained from https://cds.climate.copernicus.eu/cdsapp#!/home. Except for NASA’s AERONET data and EEA’s PM10 data, access requires prior registration. Derived dust plume indicators and obtained reconstructions are available without prior registration from https://doi.org/10.5281/zenodo.15979027.

## Code availability

The grey-scaling code can be obtained from https://github.com/tobihose/Masterarbeit. We provide the kNN implementation with the custom distance metric under https://doi.org/10.5281/zenodo.16534587.
