# Supplementary material for: Intercomparison case study of data-driven reconstructions of a cloud-obscured Saharan dust plume in Europe
Source: Sci Rep. 2026 Jun 3;16:17165. doi: 10.1038/s41598-026-55422-y (PMC13234271; doi:10.1038/s41598-026-55422-y)
Supplement: Supplementary file 1 [file 41598_2026_55422_MOESM1_ESM.pdf]

Intercomparison case study of data-driven reconstructions of a cloud-obscured Saharan dust plume in Europe - Supplementary information

Franz Kanngießer[1,2*] and Stephanie Fiedler[1,2,3,4]

[1]previously: Institute of Geophysics and Meteorology, University of Cologne, Pohligstr. 3, Cologne, D-50969, Germany.
[2]GEOMAR Helmholtz Center for Ocean Research Kiel, Wischofstr. 1-3, Kiel, 24148, Germany.
[3]Faculty of Mathematics and Natural Sciences, Kiel University, Christian-Albrechts-Platz 4, Kiel, 24118, Germany.
[4]now: Institute of Environmental Physics, Heidelberg University, Im Neuenheimer Feld 229, Heidelberg, 69120,Germany.

*Corresponding author(s). E-mail(s): fkanngiesser@geomar.de;
Contributing authors: stephanie.fiedler@uni-heidelberg.de;

References

[1] NOAA-NCEI: Federal climate complex data documentation for integrated surface data (isd). Technical report, NOAA - NCEI, US Air Force 14th Weather Squadron, 151 Patton Avenue, Asheville, NC 28801-5001 USA (January 2018)

[2] Elson, P., Andrade, E.S., Lucas, G., May, R., Hattersley, R., Campbell, E., Comer, R., Dawson, A., Little, B., Raynaud, S., scmc72, Snow, A.D., lgolston, Blay, B., Killick, P., lbdreyer, Peglar, P., Wilson, N., Andrew, Szymaniak, J., Berchet, A., Bosley, C., Davis, L., Filipe, Krasting, J., Bradbury, M., stephenworsley, Kirkham, D.: SciTools/cartopy: REL: v0.24.1. Zenodo (2024). https://doi.org/10.5281/ZENODO.13905945

[3] May, R., Arms, S., Marsh, P., Bruning, E., Leeman, J., Bruick, Z., Camron, M.D.: MetPy. UCAR/NCAR - Unidata (2016). https://doi.org/10.5065/D6WW7G29

Tab. S1 Overview over ISD codes pertaining to dust [cf. 1]

code	type	field identifier	description
07	daily present weather	AT1-AT8	dust, volcanic ash, blowing dust, blowing sand or blowing obstruction
03	present weather	AW1-AW4	haze, smoke, or dust in suspension in the air, visibility equal to or greater than 1 km
07	-	-	dust or sand raised by wind at or near the station at the time of observation, but no well-developed dust whirls(s) or sand whirl(s), and no duststorm or sandstorm seen or, in the case of ships, blowing spray at the station
3	past weather	AY1-AY2	sandstorm, duststorm or blowing snow
06	present weather	MW1-MW7	widespread dust in suspension in the air, not raised by wind at or near the station at the time of observation
07	-	-	dust or sand raised by wind at or near the station at the time of observation, but no well-developed dust whirl(s) sand whirl(s), and no duststorm or sandstorm seen or, in the case of ships, blowing spray at the station
08	-	-	well developed dust whirl(s) or sand whirl(s) seen at or near the station during the preceding hour or at the time of observation, but no duststorm or sandstorm
09	-	-	duststorm or sandstorm within sight at the time of observation, or at the station during the preceding hour
30	-	-	slight or moderate duststorm or sandstorm has decreased during the preceding hour
31	-	-	slight or moderate duststorm or sandstorm, no appreciable change during the preceding hour
32	-	-	slight or moderate duststorm or sandstorm has begun or has increased during the preceding hour
33	-	-	severe duststorm or sandstorm has decreased during the preceding hour
34	-	-	severe duststorm or sandstorm, no appreciable change during the preceding hour
35	-	-	severe duststorm or sandstorm has begun or has increased during the preceding hour
98	-	-	thunderstorm combined with duststorm or sandstorm at time of observation, thunderstorm at time of observation

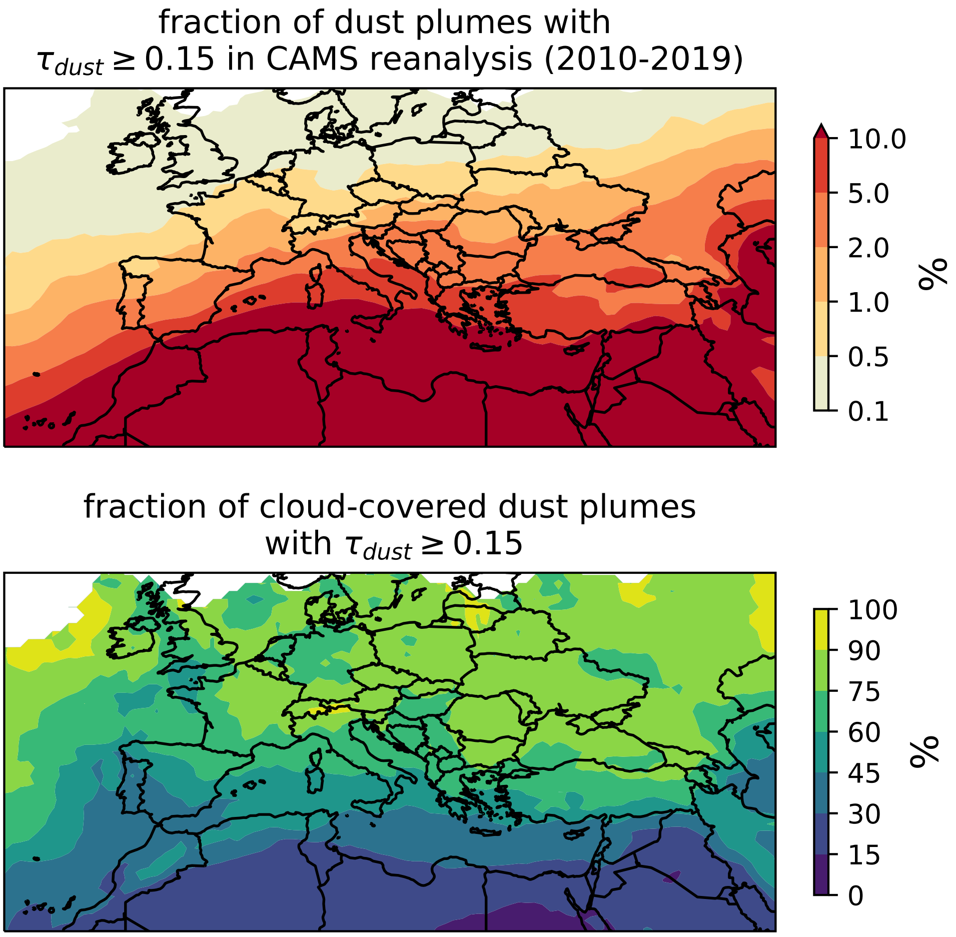

Fig. S1 The top panel shows the spatial distribution of the dust plumes in percent of time during ten years of CAMS reanalysis (2010-2019), during which a dust aerosol optical depth of at least $\tau_{\text{dust}} = 0.15$ was reached. The lower panel shows the fraction of dust plumes from the corresponding upper panel covered by clouds. Maps were created using the Python package Cartopy (version 0.24.1, https://scitools.org.uk/cartopy/docs/v0.24/index.html) [2].

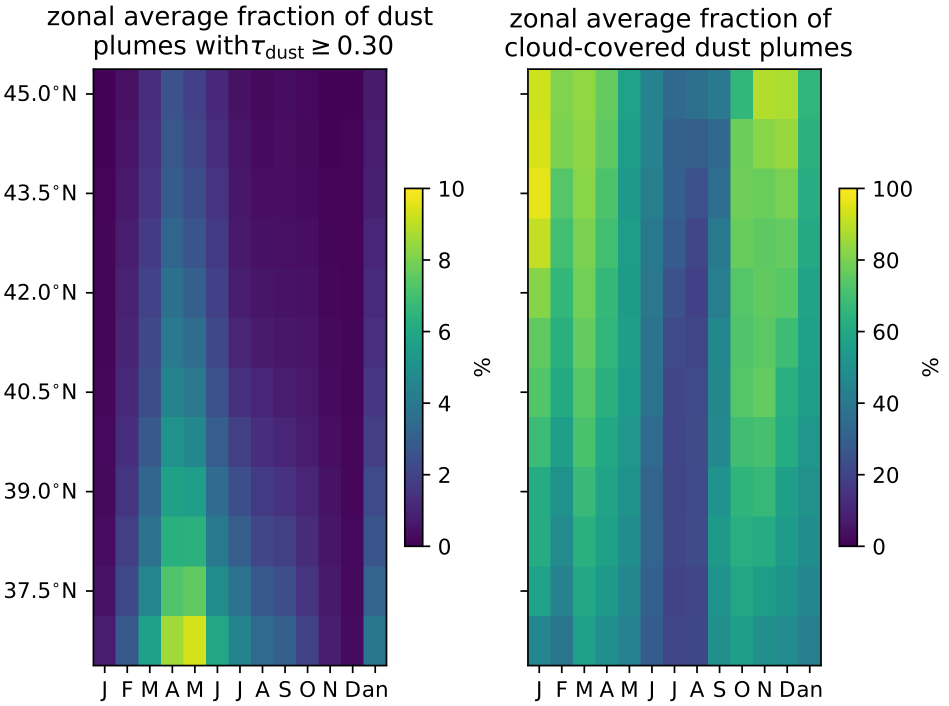

Fig. S2 Monthly and annual zonal mean temporal fraction of dust plumes in ten years of CAMS reanalysis, during which a dust aerosol optical depth of at least $\tau_{\mathrm{dust}} = 0.3$ (left) was reached and fraction of monthly and annual zonal mean fraction of dust plumes co-occurring with clouds (right).

Tab. S2 Overview over available numerical forecast models for additional cases

2022-04-23	2024-06-08	2024-06-19
ALADIN	CAMS-IFS	CAMS-IFS
CAMS-IFS	DREAM8-CAMS	DREAM8-CAMS
DREAM8-CAMS	LOTOS-EUROS	LOTOS-EUROS
EMA-RegCM4	MOCAGE	MOCAGE
LOTOS-EUROS	NCEP-GEFS	NCEP-GEFS
NCEP-GEFS	SILAM	SILAM
NOA	ZAMG-WRF-CHEM	ZAMG-WRF-CHEM
SILAM		
WRF-NEMO		

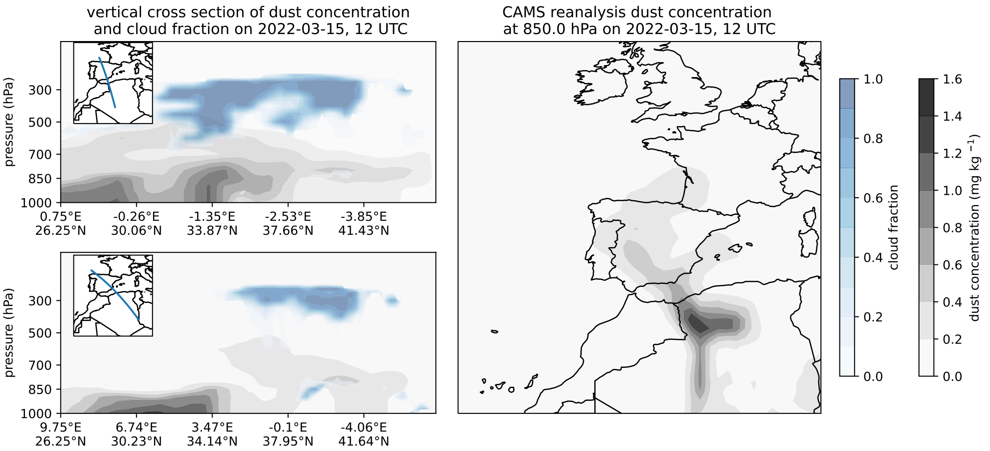

Fig. S3 Dust aerosol mixing ratio ($0.03 - 20.0\,\mu$m) from CAMS reanalysis and cloud fraction from ERA5 on 2022-03-15, 12 UTC as vertical cross-section from 26.25°N, 0.75°E to 45.00°N, 5.25°W (top left), and as vertical cross-section from 26.25°N, 9.75°E to 45.00°N, 8.25°W (bottom left). Dust concentration from CAMS reanalysis on the 850 hPa pressure level (right). Insets in the left panels indicate the location of the cross-section. Maps were created using the Python package Cartopy (version 0.24.1, https://scitools.org.uk/cartopy/docs/v0.24/index.html) [2]. The cross-sections were obtained by linearly interpolating the CAMS reanalysis in native resolution using the software package MetPy (version 1.4.1) [3].

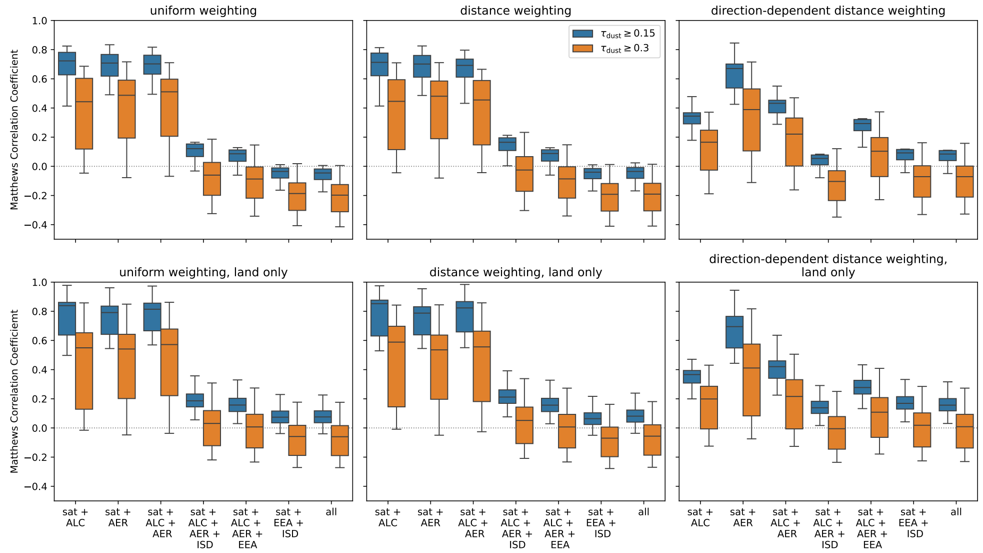

Fig. S4 MCC of data driven reconstructions with respect to spatial patterns from the numerical forecast ensemble. The different classes represent the different combinations of observational datasets. The left column shows results for applying uniform weighting in the kNN classification. The middle column displays results for kNN classifications, with points weighed according to the Euclidean distance. Results for direction-dependent distance weighting using the semi-latus rectum of an ellipse as a distance metric are shown in the right column. Directions were inferred from the SEVIRI AMV product. The top row, shows the MCC for the entire domain. The bottom row considers only grid boxes over land, since all observational datasets were land-based. For comparison, the right-most entry in each panel shows the SC of dust plumes directly extracted from SEVIRI dust RGB images with respect to the numerical forecasts.

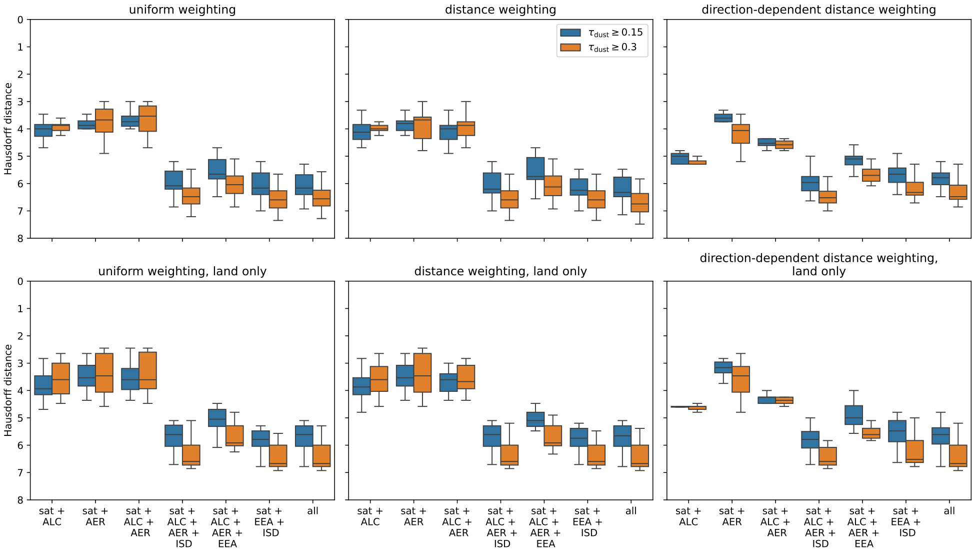

Fig. S5 As Fig. S5, but for the Hausdorff distance.

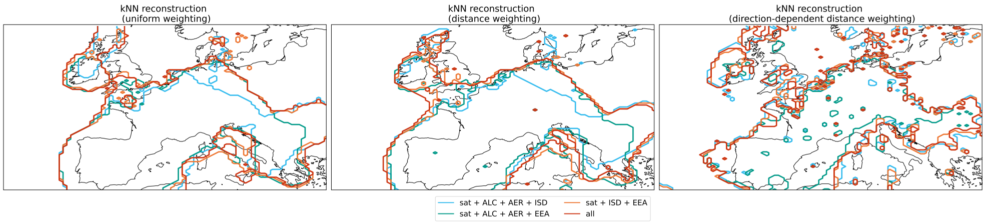

Fig. S6 Spatial patterns of dust plumes obtained from kNN reconstructions. The panels indicate different weighting of the data points, i.e. uniform weighting (left), distance weighting (centre), and direction-dependent distance weighting (right). Colours indicate the observational datasets used for the reconstruction. Only results for the four combinations of observational, resulting in the poorest reconstructions, are shown. The remaining data sets are shown in Fig. 10. Maps were created using the Python package Cartopy (version 0.24.1, https://scitools.org.uk/cartopy/docs/v0.24/index.html) [2].

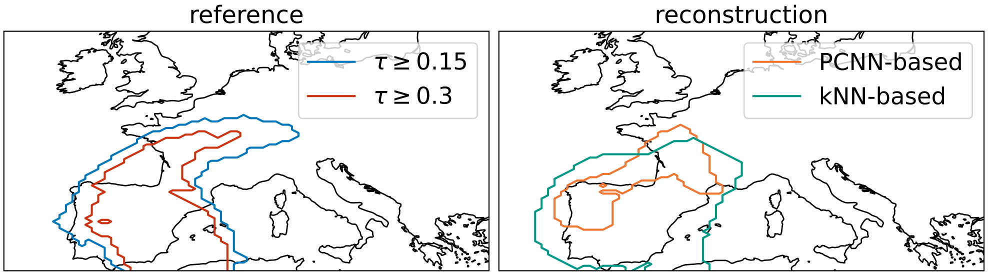

Fig. S7 Comparison of dust plumes at 12 UTC on 2022-03-15 from reference (left) and two example reconstructions (right). As reference, the areas in which at least half the operational forecast ensemble reaches the indicated values of τ. The PCNN-based reconstruction shows the results obtained with the training dataset "CAMS clim.", which has the highest SC values when compared to the full operational forecast ensemble. The kNN-based reconstruction, shown, is obtained by combining SEVIRI, AERONET, and EUMETNET ALC and using uniform weighting. This reconstruction performs best in terms of Hausdorff distance and second best in terms of SC. Maps were created using the Python package Cartopy (version 0.24.1, https://scitools.org.uk/cartopy/docs/v0.24/index.html) [2].

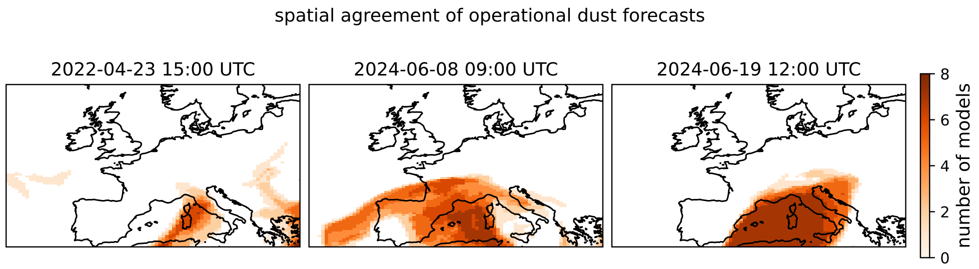

Fig. S8 Spatial agreement of individual operational forecast models in the forecast ensemble provided by the WMO Dust Regional Center Barcelona for three additional test cases, 2022-04-23 15:00 UTC (left), 2024-06-08 09:00 UTC (centre), and 2022-06-19, 12:00 UTC (right). The shading indicates the number of operational models forecasting values of $\tau_{\text{dust}} \geq 0.15$ in each gridbox. Maps were created using the Python package Cartopy (version 0.24.1, https://scitools.org.uk/cartopy/docs/v0.24/index.html) [2].

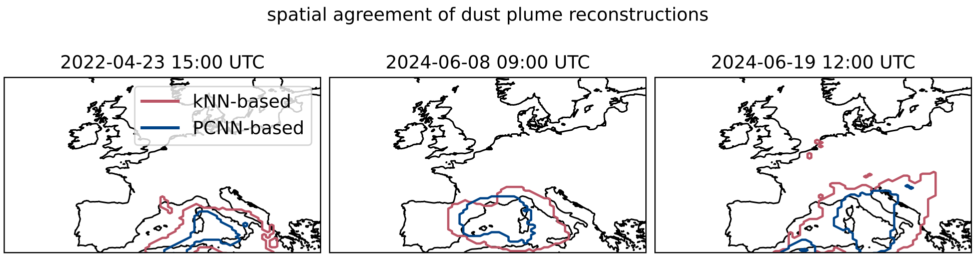

Fig. S9 Comparison of kNN-based (red) and PCNN-based (blue) reconstructions for three additional test cases, 2022-04-23 15:00 UTC (left), 2024-06-08 09:00 UTC (centre), and 2022-06-19, 12:00 UTC (right). The kNN-based reconstruction was performed by combining ground-based remote sensing data with SEVIRI information, using uniform weighting. For PCNN-based reconstructions, the network trained with the "CAMS clim." training dataset was used. Maps were created using the Python package Cartopy (version 0.24.1, https://scitools.org.uk/cartopy/docs/v0.24/index.html) [2].
